# Recent Trends in Composite Nanozymes and Their Pro-Oxidative Role in Therapeutics

**DOI:** 10.3389/fbioe.2022.880214

**Published:** 2022-05-30

**Authors:** Shilpa Maddheshiya, Seema Nara

**Affiliations:** Department of Biotechnology, Motilal Nehru National Institute of Technology, Allahabad, India

**Keywords:** nanozyme, pro-oxidative, therapeutic, antibacterial, antitumor

## Abstract

Nanozymes are inorganic nanostructures whose enzyme mimic activities are increasingly explored in disease treatment, taking inspiration from natural enzymes. The catalytic ability of nanozymes to generate reactive oxygen species can be used for designing effective antimicrobials and antitumor therapeutics. In this context, composite nanozymes are advantageous, particularly because they integrate the properties of various nanomaterials to offer a single multifunctional platform combining photodynamic therapy (PDT), photothermal therapy (PTT), and chemodynamic therapy (CDT). Hence, recent years have witnessed great progress in engineering composite nanozymes for enhanced pro-oxidative activity that can be utilized in therapeutics. Therefore, the present review traverses over the newer strategies to design composite nanozymes as pro-oxidative therapeutics. It provides recent trends in the use of composite nanozymes as antibacterial, antibiofilm, and antitumor agents. This review also analyzes various challenges yet to be overcome by pro-oxidative composite nanozymes before being used in the field.

## 1 Introduction

Nanozymes are a class of nanomaterials possessing intrinsic enzyme activity, which puts them at the center of attraction for diagnostics and therapeutic applications. Engineering of nanozymes for high catalytic activity, easy synthesis, cost-effectiveness, stability, and sometimes reusability makes them superior to natural enzymes (Haung et al., 2019; Meng et al., 2019). Ever since 2007, when ferromagnetic nanozymes were first reported to have peroxidase (POD) mimicking activity, nanozymes possessing oxidase (OXD), superoxide dismutase (SOD), catalase (CAT), esterase, or nuclease-like activities were synthesized and used in environmental or biomedical applications (Wang X. et al., 2016a; Yu et al., 2020; Yang et al, 2021; Gomma, 2021). The utilization of nanozymes in therapeutics relies on the use of their pro-oxidative and antioxidative activities. Nanozymes exhibiting POD- and OXD like properties possess the capability of converting H_2_O_2_ into reactive oxygen species (ROS) such as hydroxyl radical (•OH), singlet oxygen (^1^O_2_), and superoxide anion (•O_2_^−^) (Xiong et al., 2019; Chong et al., 2021). These radicals are highly oxidative molecules capable of interacting with proteins and lipids in the cell membrane of living cells, causing intense damage to the organelles and inducing apoptosis or necrosis-mediated cell death (Yang et al., 2020). In addition to the pro-oxidative catalytic activity, nanozymes also display antioxidative catalytic activities such as SOD and CAT, which mediate the scavenging of ROS generated in the cell and prevent cells from oxidative damage. Both these activities of nanozymes are harnessed in therapeutics such as wound disinfection, tumor therapy, nervous disorders, etc. (Yang et al., 2021). The treatment regime mediated by nanozyme-generated oxidative radicals is termed CDT (Xu et al., 2020). The use of nanozyme pro-oxidative potential has shown immense potential as nanoantibiotics for treating bacterial infections, inhibiting biofilms, wound disinfection, and healing and in tumor therapy (Wang H. et al., 2019). Despite this, their successful translation from the laboratory to the clinic is not yet achieved due to some limitations such as poor efficacy in catalyzing low concentration of H_2_O_2,_ non-reusability of nanozymes, most nanozymes are unable to engage effectively with target cells, intrinsic shortcoming of ROS due to its short lifetime (less than 200 ns), and a small diffusion distance (approximately 20 nm). In addition, the nanozyme’s non-compatibility with specific tissue environments such as highly moist wounds and weekly acidic and hypoxic tumor microenvironment (TME) restricts their practical applicability.

**FIGURE 1:**
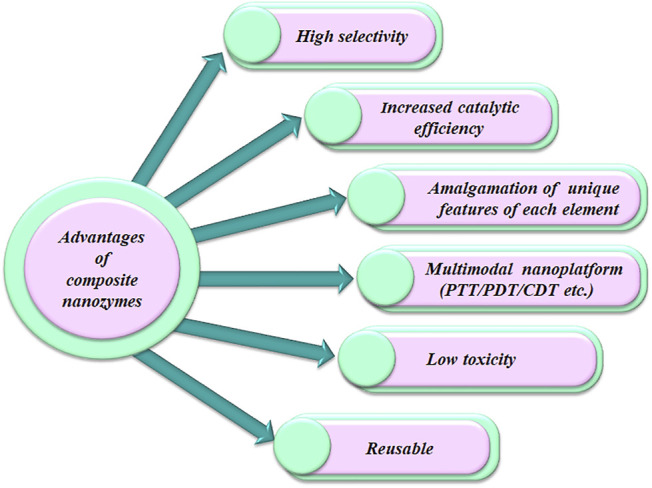
Advantages of composite nanozymes in therapeutics over other therapeutic remedies.

To circumvent such challenges, nanozymes can be engineered by tailoring their size, shape, surface, etc. One effective strategy for engineering pro-oxidative nanozymes is to produce a hybrid or alloy of more than one component. Such composite nanozymes not only inherit the individual properties of each component and overcome the shortcomings but can also integrate other functionalities (photothermal effect, photodynamic effect, etc.) to design intelligent, multifunctional nanoplatforms for disease therapy. Various advantages offered by composite nanozymes are depicted in Figure 1. Recent reviews have discussed the synthesis strategies of different nanozymes and their overall applications in diverse disciplines (Liu et al., 2020; Huang et al., 2018) or toward detection, imaging, and biomedicine development (Liang and Yan, 2019). However, focused reviews encompassing composite nanozyme-based nanoplatforms with augmented ROS capability and their applicability in therapeutics are scarce. Recently, Li Y. et al. (2021) reviewed nanozyme-based composite materials for antibacterial and antibiofilm application. Nevertheless, considering the potential embedded in the composite nanozyme-mediated ROS-based disease therapeutics and the volume of research articles published in this area, it is highly relevant to review the recent trends in this specific domain. Hence, in this review, recent advancements in designing composite nanozymes to upregulate their ROS generating potential are discussed. The strategies for making them multifunctional nanoplatforms and conferring newer properties to enhance their applicability as ROS-mediated disease treatment regimens are reviewed here. We illustrate various composite nanozymes with pro-oxidative activity used in therapeutic applications and also discuss their advantages and future prospects. We believe that this review will help in future investigations on composite nanozymes with augmented pro-oxidative potential in disease therapy.

## 2 Catalytic Activity of Nanozymes in Therapeutics

Nanotechnology has presented an opportunity to design disease therapeutics utilizing enzyme-based natural defense mechanisms in living cells such as those mediated by xanthine peroxidase, myeloperoxidase, and haloperoxidase (Chen et al., 2018). Such defense routes bestow protection by highly damaging oxide radicals against the pathogenic microorganism. Natural enzymes act as antibacterial agents by ROS production that irreversibly kills the bacteria. For example, in fresh milk, an enzyme called lactoperoxidase acts as an antibacterial agent by catalyzing H_2_O_2_ and oxidizing thiocyanate (SCN−) to produce hypothionite ions (OSCN^−^) (Davidson et al., 2015). Nanomaterials mimic natural enzymes in action and not only display pro- or antioxidative catalytic activity but also mimic hydrolase-like activity (Jiang et al.,2019). For instance, nanomaterials exhibiting DNAse (Chen et al., 2016) and phospholipase activity (Khulbe et al., 2020) cause hydrolytic cleavage of extracellular DNA (eDNA) and phospholipids, respectively, and display antibiofilm activity. Hence, a brief understanding of the mechanism of different enzyme mimetic actions of nanozymes becomes important toward designing novel nanomaterials.

### 2.1 Peroxidase Mimic Activity

**SCHEME 1:**
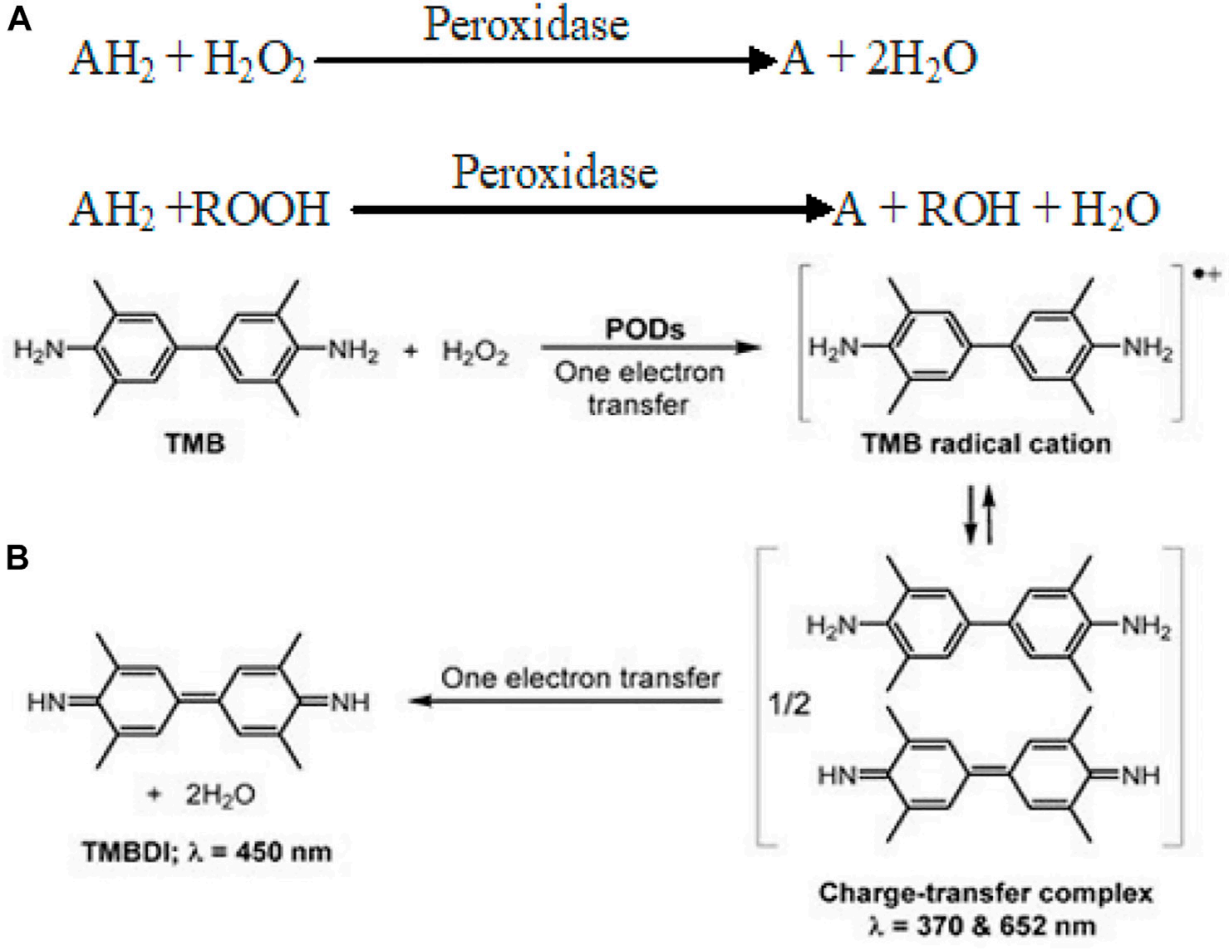
**(A)** Reaction catalyzed by peroxidase mimic nanozymes (Bhaskar et al., 2015). **(B)** Schematic for POD-catalyzed two-electron oxidation of TMB into TMBDI. Adapted and reprinted with permission from Liu et al. (2021).

Peroxidases are a group of enzymes which catalyze the oxidation of various substrates (TMB, OPD, and ABTS) in the presence of H_2_O_2_ to generate reactive-free radicals (Scheme 1A). Until now, various nanomaterials such as carbon-based, metals, metal oxides, and metal sulfides are known to display POD mimic activity (Yang et al., 2020). Fe_3_O_4_ nanoparticles were the first to demonstrate POD-like activity through a Ping–Pong kinetic mechanism (Jiang et al., 2021). POD-catalyzed oxidation of TMB/H_2_O_2_ is a two-step electron transfer process. The first step yields a TMB radical cation through single-electron oxidation. Two of these intermediate radical cations combine to form a blue-colored charge-transfer complex (= 370 and 652 nm). In the second electron transfer step, the cation radical is further oxidized to yield a TMB diimine derivative (TMBDI; λ = 450 nm). In this way, the absorption spectrum of the oxidation products of TMB usually shows three absorption bands (Scheme 1B). Following more or less similar reaction kinetics, POD mimic composite nanozymes catalyze the breakdown of H_2_O_2_ to produce toxic ROS (•OH, •O_2_) that have a promising role in therapeutics.

### 2.2 Glutathione Peroxidase Mimic Activity

**SCHEME 2:**
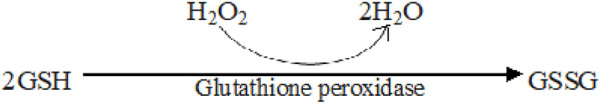
Reaction catalyzed by glutathione peroxidase mimic nanozymes (Barrington, 2013).

Glutathione peroxidase also belongs to the peroxidase family and protects organisms from oxidative damage. It oxidizes glutathione (GSH) in the presence of H_2_O_2_ to glutathione disulfide (GSSG) and H_2_O (Scheme 2). These nanozymes also exhibit a Ping–Pong catalytic mechanism wherein the nanozyme first reacts with H_2_O_2_ to form an intermediate which then oxidizes GSH to GSSG. Glutathione (GSH) is a tripeptide present in bacteria, as an antioxidant defensive system, and protects bacteria from oxidative stress by scavenging reactive-free radicals. Similarly, nanozymes with both GSH peroxidase and POD-like activity could be used as effective therapeutics in killing bacterial and cancer cells. For instance, nickel disulfide (ND) composite nanozyme with glutathione peroxidase–like activity depleted GSH and weakened the bacterial defense system, whereas its POD activity generated high ROS that irreversibly damaged bacteria (Wang et al., 2020). Similarly, the ultrasmall SnFe_2_O_4_ nanozyme possessed both POD and glutathione peroxidase activity. The SnFe_2_O_4_ nanozyme depleted GSH levels in cancer cells, and high ROS production could efficiently kill the cancerous cells (Feng et al., 2021).

### 2.3 Haloperoxidase Mimic Activity

**SCHEME 3:**
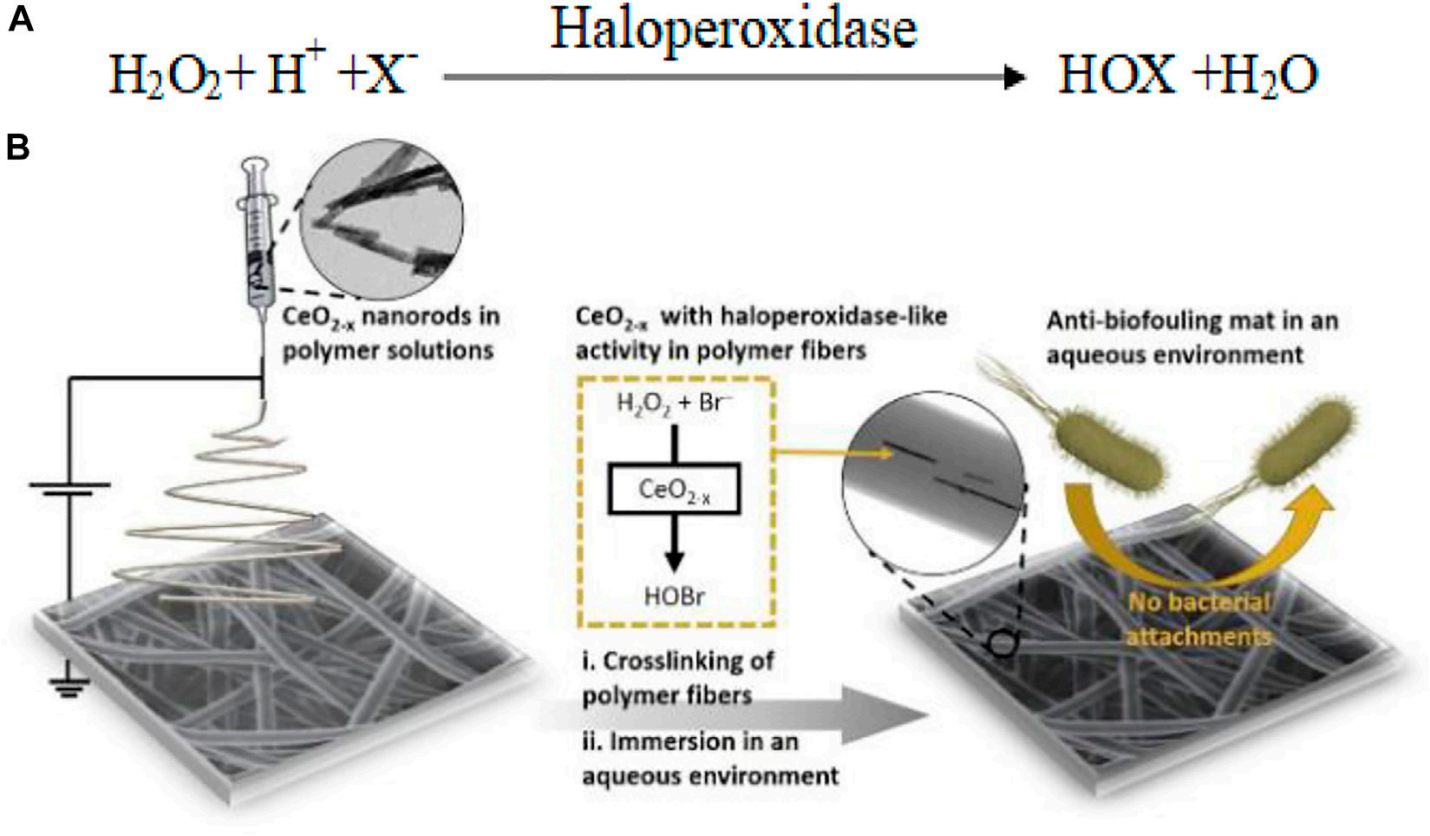
**(A)** Reaction catalyzed by haloperoxidase mimic nanozymes, **(B)** Fabrication of ceria nanofibrous mat. The hybrid mat can catalyze Br− with H_2_O_2_ to HOBr to prevent bacterial adhesion on its surface. Adapted and reprinted with permission from Hu et al. (2018), Herget et al. (2017).

Haloperoxidases also belong to the peroxidase family and catalyze the oxidation of halide ions to hypohalous acid by H_2_O_2_. Nanozyme catalytic activity has also been shown to eliminate biofilms in the marine environment. The V_2_O_5_ nanowire, with the assistance of H_2_O_2,_ converted halide ions (Cl^−^, Br^−^) to produce hypohalous acid, which caused oxidative stress to the bacteria and could protect the ships against microbial adhesion in the ocean. The local vanadium coordination geometry of the exposed lattice planes of V_2_O_5_ nanowires was similar to that of the active site of natural vanadium haloperoxidase. Vanadium atoms could function as catalytic reactive sites to produce intermediate peroxo species due to their strong affinity for H_2_O_2_, a critical step in the enzymatic reaction. As a result, the halide ions attack the more susceptible and less electron-rich oxygen atoms of the peroxo complex intermediate with the production of hypohalous acid (Scheme 3A). The strong oxidizing ability of hypohalous acid can irreversibly kill bacteria (Huang et al., 2019). Likewise, haloperoxidase mimicking CeO_2_-x nanorods (NRs) were reported to catalyze oxidative bromination of organic signaling molecules which regulate quorum sensing mechanisms in bacteria and lead to antibiofouling (Scheme 3B). This signifies the therapeutic potential of haloperoxidase mimic nanozymes for antibacterial applications (Herget et al., 2017; Hu et al., 2018).

### 2.4 Oxidase Mimic Activity

**SCHEME 4:**
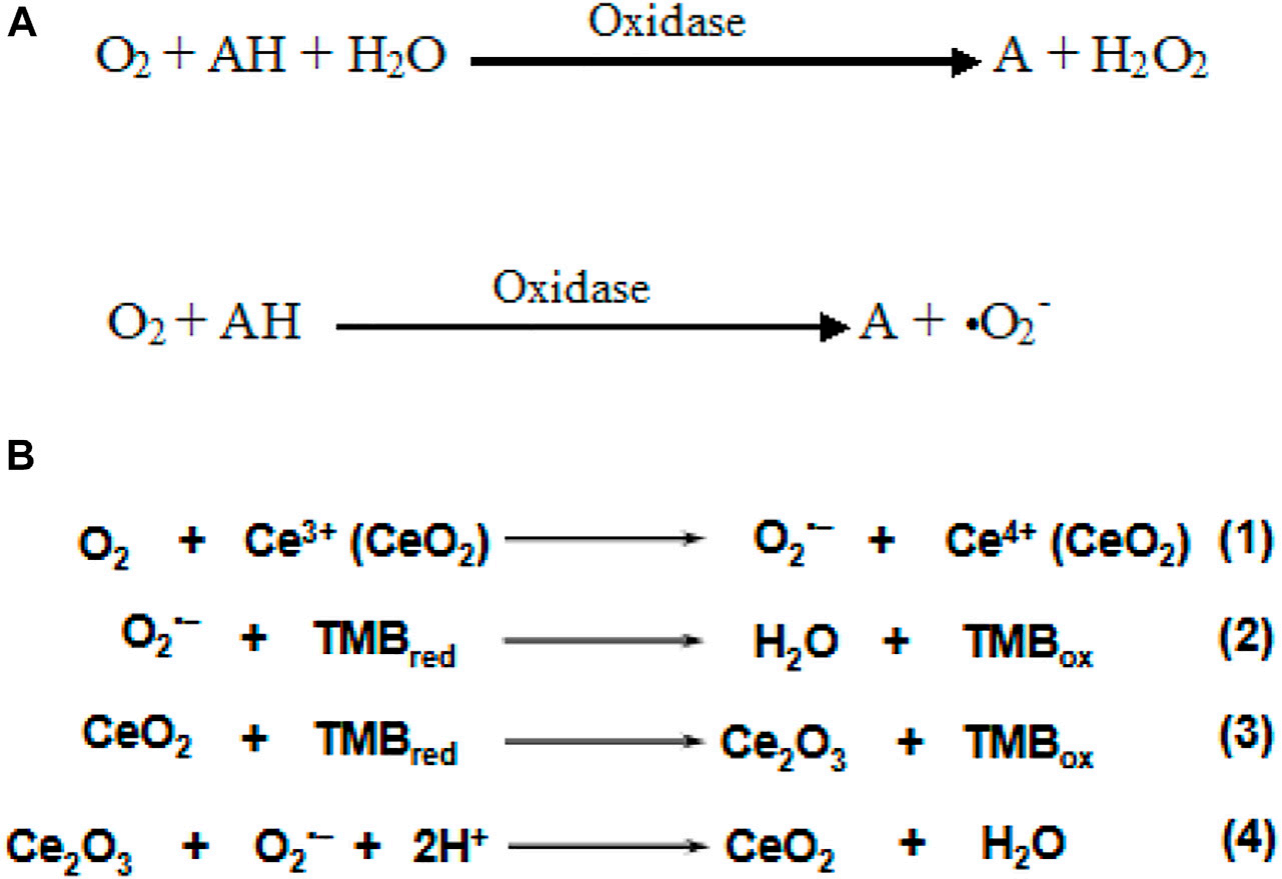
**(A)** Reaction catalyzed by oxidase mimic nanozymes (Li et al., 2020). **(B)** Equations 1–4 represent proposed mechanism of oxidase mimic nanoceria. Adapted and reprinted with permission from (Cheng et al. (2016).

Oxidase is an enzyme having a key role in cellular metabolism and processes that catalyze oxidation of various substrates by utilizing molecular oxygen to form ROS (H_2_O_2_ or •O_2_) (Scheme 4A). The oxidase family is categorized according to the acting groups of donors, including sulfur groups, amino groups, CH-OH groups, Ph-OH groups, and ferrous ions (Chong et al., 2021). Among these, the OXD-mimetic nanozymes that act on amino groups are widely studied. The generation of intermediates (e.g., singlet oxygen, oxygen superoxide anion) and the electron transfer process have a significant effect on the OXD mimic property of nanozymes. Cheng et al. reported OXD-mimic activity of nanoceria where O_2_ molecules are first adsorbed onto defect-rich sites of nanoceria and converted into O_2_^**·**−^ under acidic conditions (Eq. 1). Subsequently, TMB was oxidized and Ce^4+^ present on the surface was reduced to Ce^3+^ (Eq. 3). As the main intermediate, the *in situ* produced O_2_^**·**−^ finally regenerated Ce^4+^ *via* the oxidation of Ce^3+^, accompanied by the generation of water (Eq. 4). Alternatively, the oxidation of TMB could be directly initiated by O_2_^**·**−^ as well (Eq. 2) (Cheng et al., 2016). Recently, various nanostructures displaying OXD activity were reported to have potential application in therapeutics. Wang et al. designed Co_4_S_3_/Co(OH)_2_ HNTs to mimic OXD-like activity that can catalyze O_2_ to generate ROS such as •O_2_. It displays excellent antibacterial properties against gram-negative bacteria (*E. coli* and *P. aeruginosa*) and gram-positive bacteria (*S. sciuri* and *Bacillus*) and avoids H_2_O_2_ toxicity (Wang et al., 2020).

### 2.5 DNase Mimic Activity

**SCHEME 5:**
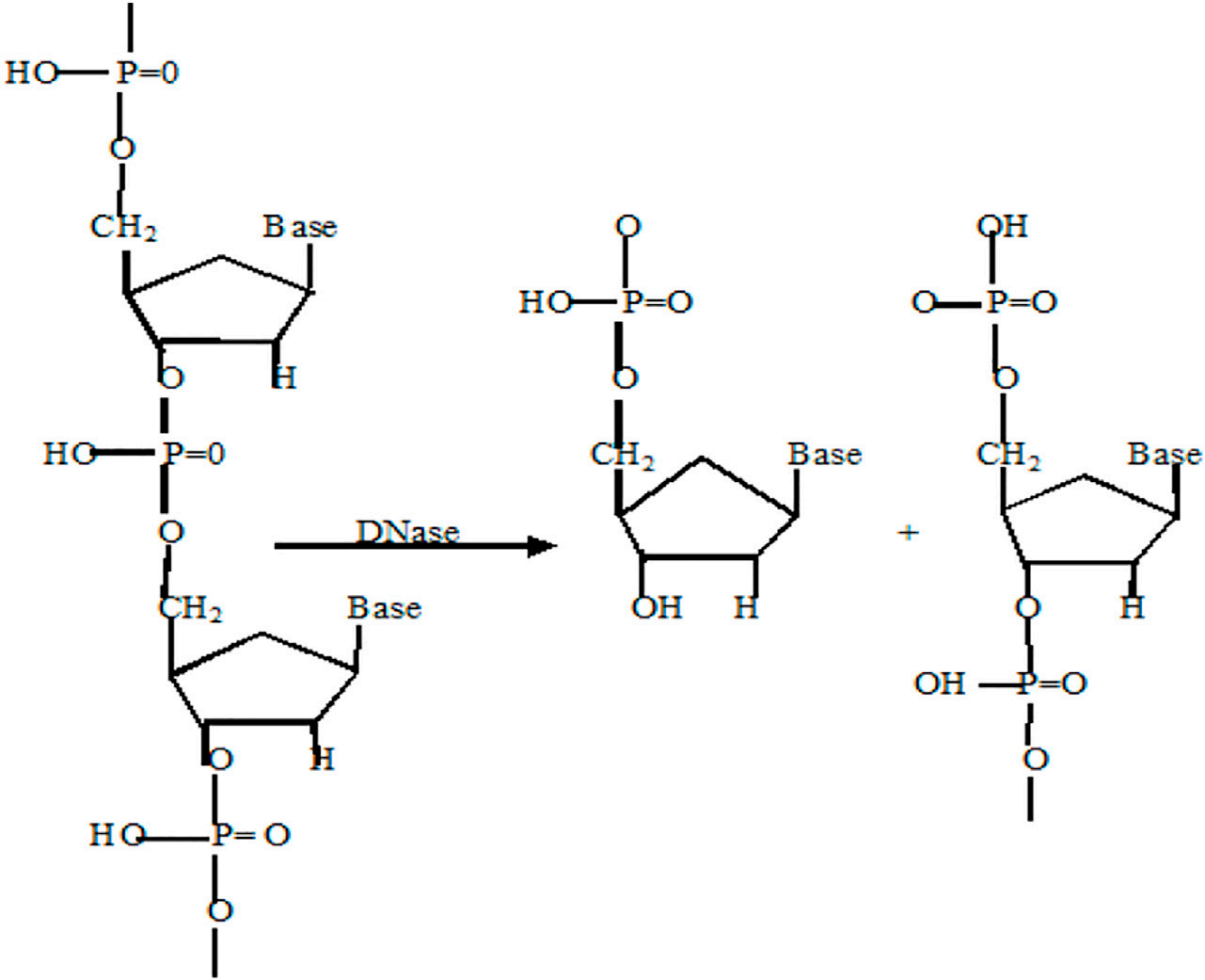
Reaction catalyzed by DNase mimic nanozymes (Laukova et al., 2020).

Deoxyribonuclease (DNase), a type of nuclease, cleaves phosphodiester linkage in DNA molecules and degrades it into fragments (Scheme 5). DNases can be used for gene editing, DNA repair, or as antibiotics in the treatment of skin diseases and bacterial infections (Fang and Liu, 2020). A DNase-mimetic artificial enzyme (DMAE) synthesized by incorporating cerium (IV) ion complexes on the Au component of Fe_3_O_4_/SiO_2_ core/shell nanoparticles showed excellent DNase-like activity against bacterial biofilms. With its greater penetration inside the biofilm, DMAE caused 80% eDNA cleavage within the biofilm and prevented upto 90% bacterial adhesion (Chen et al., 2016). Recently, it was reported that the combination of POD and DNAse activity in a single nanozyme (MOF-2.5Au-Ce) could achieve total elimination of the biofilm and prevention of secondary biofilm formation (Liu et al., 2019).

### 2.6 Phospholipase Mimic Activity

**SCHEME 6:**
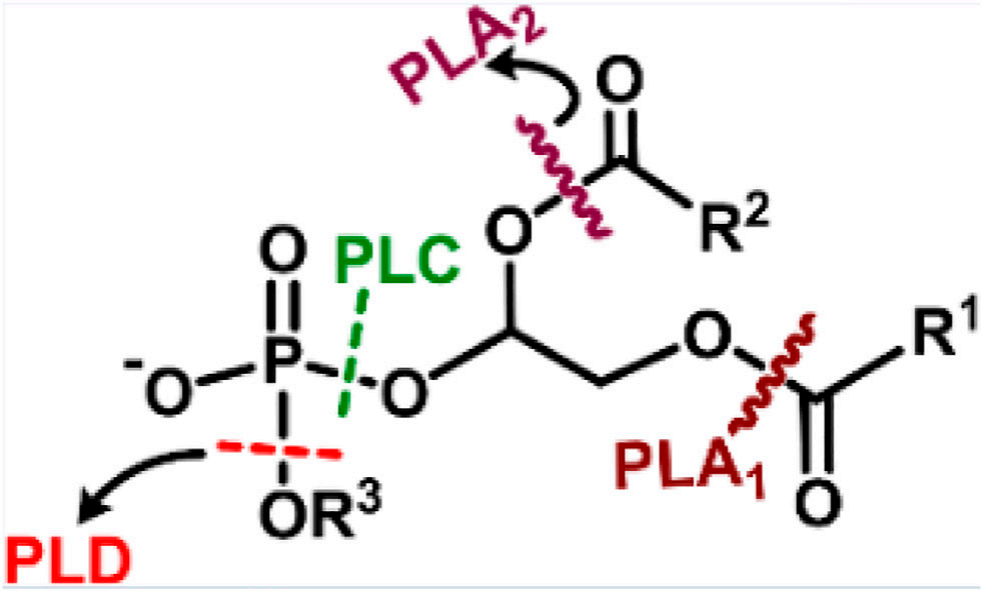
Depiction of various cleavage sites of a phospholipid by different phospholipases, **PLA**_**1**_-cleaves sn-1 acyl chain; **PLA**_**2**_-cleaves the sn-2acyl chain; **PLC**-cleaves before phosphate, releasing diacylglycerol and phosphate-containing group; **PLD**- cleaves after phosphate, releasing phosphatidic acid and alcohol. Adapted and reprinted with permission from Khulbe et al. (2020).

Phospholipase is an enzyme that catalyzes the hydrolytic cleavage of phospholipids at various sites (Scheme 6). A major component of the bacterial cell membrane is phospholipid, which plays a key role in biofilm formation, and its cleavage could disrupt biofilms. Phospholipase-mimetic ceria-based nanozyme could hydrolyze the phospholipids in bacterial cell membranes and bacterial biofilms efficiently (Khulbe et al., 2020). The study also demonstrated the potential of nanozyme in preventing bacterial colonization on the surface of urinary catheters. However, scarce literature is available for applying phospholipase mimetic nanozymes for therapeutic applications.

Table 1 presents a comparison of the aforementioned enzyme mimic activities displayed by nanozymes that kill bacteria or cancerous cells through different mechanisms. Some of these such as DNAse and phospholipase mimic nanozymes are relatively less explored. The design of a nanozyme that could exhibit and utilize more than one type of these pro-oxidative activities to kill its target cell may have high potential as a therapeutic agent.

**TABLE 1:** Comparison of various pro-oxidative enzyme mimic activities exhibited by composite nanozymes.

Enzyme activity displayed	Nanozyme mimic	Substrate	Mechanism of action	Therapeutic applications
Peroxidase	Broad range including metal, metal oxide, metal organic framework–based, and carbon-based nanozymes	H_2_O_2_	Generated ROS (•OH, •O_2_) cause oxidative stress–mediated cell killing	Antibacterial, anticancer, antibiofilm, and wound healing
Oxidase	Most nanoparticles such as Au, NiO, Pd, V_2_O_5_, IrOx, etc	O_2_	Generates ROS (H_2_O_2_ or •O_2_) that causes oxidative damage	Antibacterial; anticancer
Glutathione peroxidase	Few nanoparticles such as NiS_2,_ PdFe/GD	Glutathione and H_2_O_2_	Deplete glutathione by converting into glutathione disulphide and weakens bacterial defense system	Antibacterial; anticancer
Haloperoxidase	Nanoparticles such as CeO_2_ and V_2_O_5_	Halide ions (Cl ^and^, Br^−^)	Generate hypohalous acid which causes oxidative cell damage	Antibiofilm; antibiofouling
DNase	Nanoparticles such as CeO_2_ and MOF/Ce	DNA	Cleaves DNA of biofilm into fragments	antibiofilm
Phospholipase	Nanoparticles such as Nanoceria	Phospholipids	Hydrolysis of long-chain phospholipids present on the bacterial cell membrane and disrupt it	antibiofilm; antibacterial

## 3 Composite Nanozymes With Pro-oxidative Potential

**FIGURE 2:**
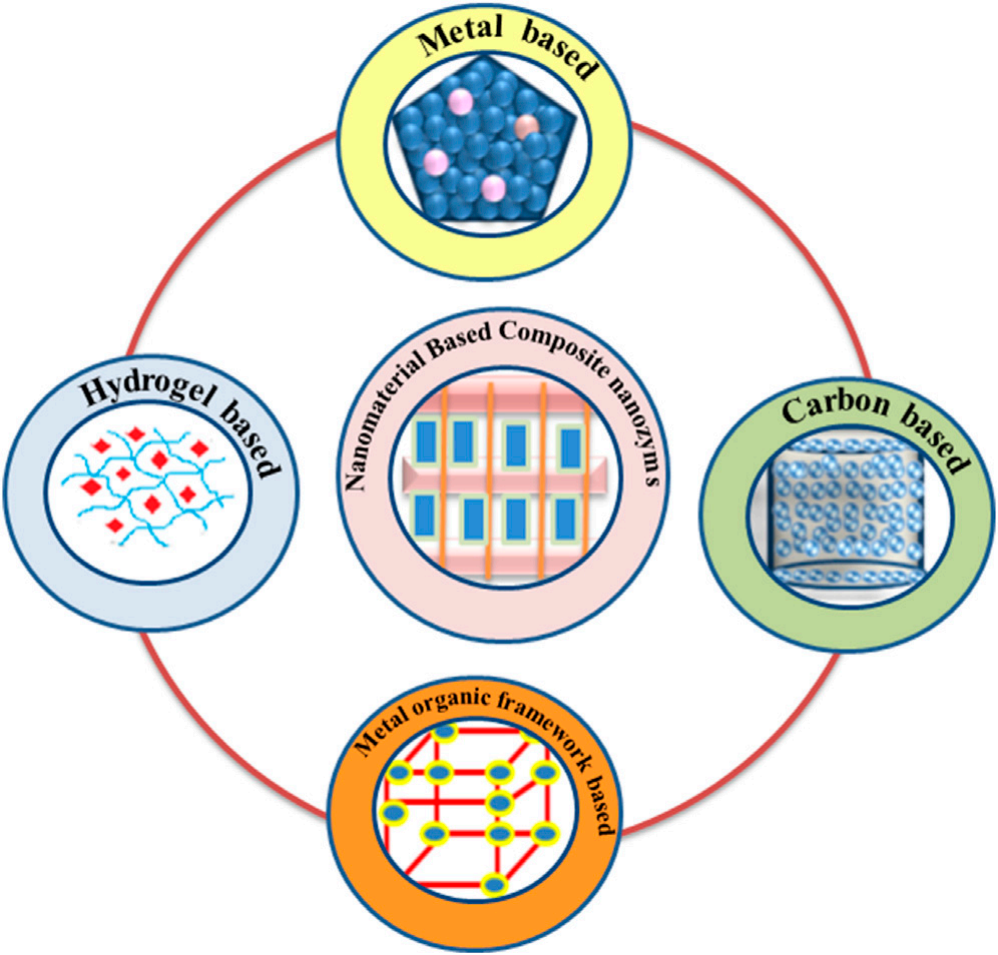
Nanomaterial-based composite nanozymes with pro-oxidative enzyme-like activity.

Nanozymes, particularly new-generation hybrid nanostructures, have aroused increasing attention by virtue of their superior pro-oxidative potential compared to natural enzymes and have shown practical applications such as antibacterial, antibiofilm, and antitumor in therapeutics. Currently, a number of hybrid or composite nanozymes (Figure 2) are being designed by integrating their pro-oxidative potential with other functionalities and using them as multifunctional nanoplatforms in disease therapeutics as discussed here.

### 3.1 Metal-Based Composite Nanozymes

Metal-based nanoparticles are well known to mimic POD and OXD activity, with promising applications in therapeutics. Metal-based nanozymes are in vogue owing to their easy synthesis and facile surface modification and convenience to hybridize with other elements and tailor their shape-size, high stability, biocompatibility, and electromagnetic functionality (Li M. et al., 2020; Shi et al., 2020). For instance, Xi et al. have shown that the doping of Cu in hollow carbon spheres (Cu-HCS) accelerated their POD activity (Xi et al., 2019). The catalytic activity of the nanohybrid was dependent on the state of the metal (Cu^0^/Cu^2+^) rather than its content or size as CuO-HCS displayed eight times lower catalytic activity than Cu-HCS, despite a very high Cu concentration in the former. Moreover, CuO-HCS nanozymes showed antibacterial activity against only Gram-negative bacteria in the absence of H_2_O_2_ through the release of Cu^2+^ ions, whereas Cu-HCS displayed broad-spectrum antibacterial activity at low H_2_O_2_ concentrations through ROS generation. Metallic nanocomposites such as PEG-Cu_2_Se HNCs nanozyme was used as a multifunctional nanoplatform for antitumor application. It displayed enhanced antitumor activity due to synergistic effects of Fenton-like reactions with CDT and PTT without any long-term toxicity (Wang et al., 2019). Noble metals (Ag, Au, Pd, and Pt) exhibit strong catalytic activity, which have been used for designing nanohybrids and have application in cancer therapy, immunological assays, and antibacterial agents (Cai and Yang, 2020; Sharifi et al., 2020). Bifunctionalized mesoporous silica-supported gold nanoparticles (MSN-Au NPs) displayed dual-enzyme activity (POD and OXD) to generate ROS such as • OH, ^1^O_2,_ and •O_2_^−^. As a result, the MSN-Au NPs exhibited effective antibacterial and antibiofilm properties (Tao and Ju, 2015). Mirhossein et al. fabricated core-shell Au@Co-Fe hybrid nanoparticles (Au@Co-Fe NPs) in which all nanoparticles such as Au, Fe, and Cu possess POD activity. Thus, these NPs in combination displayed enhanced enzyme activity and exhibited ROS-mediated excellent antibacterial activity against *E. coli, P. aeruginosa*, *S. aureus*, and *B. cereus* (Mirhosseini et al., 2020). Along with antibacterial activity, Au nanozyme also acts as an imaging agent and can be used for imaging infections associated with the biofilm. Therefore, a hybrid of AuNPs provides new scope for diagnosis and therapeutic applications for bacterial diseases. Ag nanoparticles provide good optical response and are easily miscible in any alloy composition. Hence using Ag with Pd nanoparticles with POD mimic activity could prove to be promising for tumor treatment. Li et al. demonstrated that AgPd@BSA/DOX nanocomposite possessed enhanced POD-like activity and better photothermal conversion efficiency (PCE) under NIR light irradiation as than individual Ag or Pd metal. Ag/Pd nanocomposite also functioned as a nanovehicle for carrying doxorubicin drugs, which were released under NIR light, induced hyperthermia and by a synergistic mechanism, and acted as an effective therapeutic agent. It was demonstrated that AgPd@BSA/DOX could catalyze conversion of H_2_O_2,_ which is overexpressed in the TME, to generate a high amount of •OH, causing cell cycle arrest of cancerous cells, apoptosis, and senescence (Li L. et al., 2020). Another bimetallic nanozyme silk fibroin capped AuPt responded to tumor hypoxia and its reducing environment to treat tumors with high efficiency. AuPt@SF (APS) decomposed glucose to produce abundant H_2_O_2_ and oxidized intratumoral GSH to resist ROS depletion and simultaneously catalyzed O_2_ and H_2_O_2_ to generate ROS such as superoxide radicals (•O_2_^−^) and hydroxyl radicals (•OH), respectively (Yang W. et al., 2021a).

### 3.2 Metal Oxide and Sulfide Based Nanozymes

Recently, various metal oxides such as Fe_3_O_4_ (Fu et al., 2017), MnO_2_ (Liu et al., 2021), and CeO_2_ (Zhu et al., 2021) with significant pro-oxidative enzymatic activity have been utilized for therapeutic applications. Among different metal oxide–based nanozymes, Fe_3_O_4_ nanoparticles (NP) received great attention owing to their excellent POD-like activity. Vallabani et al. constructed an ATP-triggered citrate-coated Fe_3_O_4_ NP with POD mimetic activity. Generally, most nanozymes are active under an acidic condition toward H_2_O_2_ breakdown, and this limits the practical application of nanozymes at physiological pH, but in this nanoalloy, ATP formed a complex with Fe^2+^ in the presence of H_2_O_2_ and generated ROS such as •OH, which promoted chronic wound healing at pH near 6.5–8.5. This nanocomposite displayed high antibacterial/antibiofilm activity (Vallabani et al., 2020). Karim *et al.,* found that photoactive CuO nanorods (NRs) with POD-mimetic activity could act as a potent antibacterial agent. The catalytic activity increased approximately 20-fold due to strong affinity with H_2_O_2,_ producing large amounts of •OH at a low concentration of H_2_O_2_ molecules (Karim et al., 2018). Wei et al. fabricated a defect-rich rough surface Fe_3_O_4_@MoS_2_-Ag composite nanozyme with POD mimicking activity. Fe_3_O_4_@MoS_2_-Ag generated ROS and leaked Ag^+^ killed ∼69.4% *E. coli* cells. It also displayed photothermal response under NIR activation to achieve outstanding synergistic disinfection (∼100%). The magnetic properties of Fe_3_O_4_ made it feasible to recycle it (Wei F. et al., 2021).

Currently, manganese dioxide (MnO_2_), a transition metal oxide, in combination with other components, is widely explored for cancer therapy with special and unique physicochemical properties. The MnO_2_ nanostructure is highly sensitive to the TME and rapidly degrades in the reduced and acidic environment, which is why it can be used as a tumor-specific drug vehicle. In addition, MnO_2_ is shown to catalyze H_2_O_2_ overproduced in tumor cells to produce O_2_ *in situ* and overcome TME hypoxia (Wu M. et al., 2019b; Zhu et al., 2020). Zhu et al. constructed a composite core–shell-structured nanozyme (MS-ICG@MnO_2_@PEG) having indocyanine green (ICG) loaded as a photosensitizer in the MnO_2_ shell for photodynamic therapy (PDT) clubbed with ROS-mediated chemodynamic therapy (Zhu et al., 2020). MnO_2_ also catalyzed intratumoral glutathione (GSH) to convert Mn^4+^ oxidation state into Mn^2+^ oxidation state, which simultaneously decomposed H_2_O_2_ by POD-like activity to form highly reactive •OH. Mn^2+^ being water-soluble were excreted easily from the body without causing toxicity**.** Li et al. fabricated MnO_2_/IrO_2_-PVP nanocomposite and loaded it with a photosensitizer Chlorin e6 (Ce6) that specifically responded to the TME. MnO_2_ in tumors catalyzed the production of O_2_ by H_2_O_2_ to alleviate hypoxia condition and also reacted with H^+^ and performed MRI function, whereas IrO_2_ possessed photothermal activity that converted O_2_ formed in the tumor to toxic singlet oxygen upon light irradiation, thereby enhancing the PDT. Thus MnO_2_/IrO_2_-PVP nanocomposite by synergistic mechanism displayed outstanding antitumor therapy (Li J. et al., 2021).

In recent years, experiments indicate that metal-sulfide nanomaterials with enzyme-like activity exhibit excellent antibacterial properties. Xu et al. reported a strategy for converting garlic-derived natural organosulfur compounds into a nano-iron sulfide that exhibited excellent antibacterial activity. It was shown that nano-iron sulfide with POD- and CAT-like activities can catalyze the oxidation of H_2_O_2_ to generate highly toxic hydrogen polysulfide. Nano-iron sulfide exhibited 500-fold increased antibacterial efficacy and also eliminated biofilms on human caries and promoted wound healing (Xu et al., 2018). Yin et al. synthesized molybdenum disulfide nanoflowers functionalized with polyethylene glycol that possessed high POD and PTT activity under NIR absorption. PEG-MoS_2_ NFs generated high ROS and disrupted the membrane of drug-resistant and endospore-forming bacteria and promoted wound healing (Yin et al., 2016).

### 3.3 MOF Material–Based Composite Nanozymes

The metal-organic framework (MOF) is a type of porous crystalline material designed by incorporating metal-containing nodes with organic ligands linked through coordination bonds (Wang et al., 2020; Ding S. et al., 2020). The different types of metal nodes/organic ligands with tailorable hollow cavities and open channels in MOFs provide high surface area, ease of pore tuning, property adjustability, and structural diversity. Facile modification of metal and ligand makes MOFs a promising host for immobilizing various metals, metal oxides, quantum dots, biomacromolecules, etc. (Zhao W. et al., 2019; Chen et al., 2018). Similar to natural enzymes, MOF cavities provide a hydrophobic environment; ordered arrangement of active catalytic sites offers highly dense substrates and mimics the enzyme activity (Lee et al., 2009; Cai and Yang, 2020). MOF-based composite nanozymes with various functional ligands have been fabricated to generate either ROS or reactive nitrogen species (RNS) for highly efficient antibacterial therapy. Cheng et al. designed biomimetic L-Arg/GOx@CuBDC with glucose oxidase (GOx), POD, and nitric oxide synthetase mimetic activity as an excellent antibacterial agent (inactivation efficacy ≥97%) for both gram-positive and gram-negative bacteria. The mechanism of L-Arg/GOx@CuBDC action was based on a double-cascade reaction system which under aerobic conditions first converted endogenous glucose to gluconic acid and H_2_O_2,_ which acted as an initiation reaction for generating ROS. In the case of the RNS cascade reaction, NO produced by oxidation of L-Arg in the presence of H_2_O_2_ quickly reacted with •O_2_^−^ (ROS) to generate ONOO^−^ which was highly toxic (Cheng et al., 2020). Ma et al. designed IL@MIL-101(Fe)@BSA-AuNCs nanocomposite NPs mimicking POD-like activity and also possessed dual-modality imaging properties. Under microwave irradiation, MIL-101 (Fe) catalyzed H_2_O_2_ to generate •OH and treat tumor cells with high efficiency. The BSA-Au NCs were used for detecting dynamic distribution processes and diagnosing tumor sites with high specificity (Ma et al., 2019).

### 3.4 Carbon-Based Nanozymes

Carbon-based nanomaterials that possess catalytic activities include graphene, carbon nanotubes, fullerene, and carbon dots (Sun H. et al., 2020), and their derivatives find wide applications in diverse fields due to their outstanding electronic, optical, thermal, mechanical properties, low cost, biosafety, and multienzyme mimicking activities (Wang X. et al., 2016b; Ding H. et al., 2020). Fan et al. fabricated a nitrogen-doped porous carbon nanosphere mimicking multienzyme activity to target and destruct tumor cells with very high efficacy. Here, ferritin was integrated to guide nanozyme for specifically targeting HFn receptor (TfR1)–positive tumors and killing tumor tissue through abundant ROS generation. More importantly, the porosity of the nanocomposite provided numerous active sites for the substrate and improved and enhanced the catalytic activity (Fan K. et al., 2018). Graphitic carbon nitride nanosheets (g-C_3_N_4_ NSs), a carbon-based nontoxic semiconductor, exhibited POD mimetic activity and showed good potential as an antibacterial. The nanocomposite g-C_3_N_4_@Au NPs with POD mimetic activity were prepared by integrating Au NPs which stabilized the free radical and exerted a positive synergistic coupling effect. They showed potent antibacterial activity against multidrug resistant *S. aureus* and antibiofilm activity in the presence of a low amount of H_2_O_2_. In addition, the g-C_3_N_4_@Au Band-Aid was designed to combat *in vivo* bacterial infection and promote wound healing in the presence of ultralow concentrations of H_2_O_2_ (10 µM**)** (Wang et al., 2017). An alloy of carbon nanozymes also displayed photothermal activity under NIR and not only acted as an antibacterial agent but also enhanced their catalytic activities. Xi *et al.* fabricated N-doped sponge-like carbon spheres (N-SCSs) with multienzyme mimetic activity and photothermal response for synergetic antibacterial therapy. Under NIR, the catalytic activity of N-SCSs increased due to the laser irradiation that exposed a more active surface. When bacteria were kept with N-SCSs for 20 min and then irradiated with NIR light again for 10 min, the antibacterial performance increased toward *E. coli* (>−3.0 Lg (CFU ml^−1^)) and *S. aureus* (*>*−2.0 Lg (CFUml^−1^) (Xi et al., 2019).

### 3.5 Hydrogel-Based Nanozymes

Hydrogels are known to maintain a moist environment and could act as a barrier for microbes around the wound interface. Newer techniques have been used to integrate ROS-generating composite nanozymes with hydrogels for sustained and efficient therapeutic applications. A PVA hydrogel incorporated with rGO/MoS_2_/Ag_3_PO_4_ composites was synthesized to have enhanced photothermal and photocatalytic function and was used for rapid and effective treatment of bacterial infection in chronic wound healing (Zhang et al., 2019). The mechanical property and swelling ratio of hydrogels were significantly improved with rGO. In another study, ZnO QDs@GO NCs were introduced into chitosan hydrogels and designed as a multifunctional platform. Zn^2+^ produced with the dissolution of ZnO QDs by lysosomal acid was absorbed by bacteria, leading to inhibition of respiratory enzymes and ROS generation, whereas GO acted as a photosensitizer under NIR irradiation. This multifunctional hydrogel showed excellent wound healing and antibacterial applications in moist conditions (Liang et al., 2019). Similarly, poly-2-dimethylaminoethyl methacrylate (PDMAA) hydrogels were decorated with multifunctionality by encapsulating ROS generating hollow carbon nanoparticles and aloe-emodin (AE antibiotic extracted from Aloe leaves) within them**.** NIR-triggered ROS generation caused immediate bacterial killing, and continuous release of AE from the gel showed long-term effects and accelerated recovery of an infected wound (Xi et al., 2018).

Excessive amounts of free copper for therapeutic applications induced toxicity, which could be reduced with the utilization of hydrogels. Qiu et al. constructed the hydrogel-based artificial enzyme comprising copper and amino acids with good biocompatibility and peroxidase mimetic activity, exhibiting broad-spectrum antibacterial activities against both drug-resistant Gram-positive bacteria and Gram-negative bacteria. Furthermore, this system was prepared to function as a wound dressing, which could combat wound pathogens effectively and promote wound healing by stimulating angiogenesis and collagen deposition (Qiu et al., 2020). Sang et al**.** reported that MoS_2_-hydrogel mimicking peroxidase nanozyme could efficiently capture bacteria and realize excellent antibacterial and wound healing efficiency compared to traditional nanozyme. In addition, the system removed dead bacteria from the wound and reduced the incidence of inflammation (Sang et al., 2019). A comparison of the advantages and shortcomings of the abovediscussed classes of composite nanozymes is compared in Table 2.

**TABLE 2:** Advantages and disadvantages of different composite nanozymes.

Composite nanozymes	Advantages	Disadvantages
Metal-based	Easy synthesis, facile surface modification, tunability of shape-size, electromagnetic functionality, high catalytic activity, easy to hybridize with other elements, positive synergistic coupling effect, and ability to stabilize free radicals (Au NPs)	Only few metals are biodegradable and releases in the form of ions cause toxicity
Metal-oxide based	High stability, easy to prepare, adjustable porosity, facile incorporation into hydrophobic and hydrophilic systems, and good redox chemistry	Toxic, traditional method of synthesis is not feasible
Metal-organic framework–based	Porous structure provide abundant surfaces and channel for electron transfer, adsorption, loading, and separation of targets, metal nodes in MOF provide the possible active sites for catalysis, and organic ligands offer rich functional groups for chemical modification	Toxic, poor selectivity, and difficulties in recycling and regeneration
Carbon-based	Excellent transportation property (e.g., graphene), rich surface chemistry, low cost non-toxicity (graphitic carbon nitride), long-term storage, and high stability	Low catalytic activity, catalytic mechanism unknown, difficulties in rational design, and construction
Hydrogel- based	Flexile, biocompatible, capture target cells with high efficiency due to charge and pore. Biodegradable, diversiform structure and properties, easily transportable, and easy to tune	limited nanozyme type, low mechanical strength, and difficult to handle and are expensive, non-adherent

## 4 Engineering Composite Nanozymes for Enhanced Pro-oxidative Activity

**FIGURE 3:**
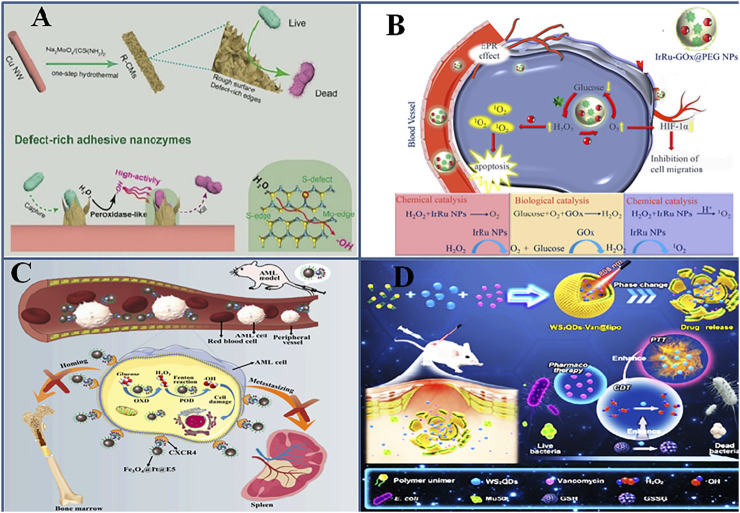
Engineering composite nanozymes with enhanced pro-oxidative **(A)**. Defect-rich surface of composite nanozymes enhance the affinity of composite nanozymes to bacteria. Adapted and reprinted with permission from reference (Wang et al., 2020). **(B).** Multicatalytic action of composite nanozymes perform cascade reaction and exert superior activity. Adapted and reprinted with permission from the reference (Wei et al., 2020) **(C)**. Conjugation of CXCR4 to Fe_3_O_4_@Pt@E5 specifically target cancerous cells and synergistically treat AML (Kong et al., 2021) **(D).** Multifunctionality such as photothermal/chemodynamic/pharmaco of WS_2_QDs synergistically kill and eradicate bacteria Adapted and reprinted with permission from reference (Xu et al., 2020).

Composite nanozymes can be variously tailored to generate strong ROS that can be subsequently used in antitumor, antibacterial, or wound healing applications. A number of factors can be considered to design an effective composite nanozyme with strong pro-oxidant potential (Figure 3).

### 4.1 Choice of Materials

The choice of materials used to synthesize hybrid or composite nanozymes with high ROS generating ability is highly crucial. H_2_O_2_ is considered an effective antibacterial disinfectant. However, a biologically safe level of H_2_O_2_ can only be used for generating •OH radicals. Hence, it is imperative to design highly efficient catalytic nanozymes that can catalyze the conversion of very low levels of H_2_O_2_. Composite nanozymes can solve this problem to a great extent. Some materials such as AuNPs exert a synergistic coupling effect by generating more ROS and stabilizing the generating radicals. For instance, Wang et al. integrated AuNPs with g-C_3_N_4_ because -NH_2_ and -NH groups on g-C_3_N_4_ nanosheets served as effective Lewis bases to chelate metal ions and showed high affinity for Au^3+^, leading to the growth of AuNPs on g-C_3_N_4_ nanosheets. The composite nanozyme possessed excellent peroxidase ability as compared to only AuNPs and g-C_3_N_4_ nanosheets, which was due to the stabilization of •OH radicals generated by the breakdown of H_2_O_2_ by AuNPs *via* partial electron exchange interaction (Wang et al., 2017). Ultrasmall AuNPs possess high catalytic activity but are prone to aggregation, which can be reduced by producing hybrids with 2D MOFs as the latter can impose fast kinetics and effectively lower mass-transfer resistance of catalytic reactions (Hu et al., 2020).

The adsorption energy of metallic NPs dispersed on any solid support and the electron transfer capability of the nanocomposite greatly influences their catalytic ability. Wang et al. prepared PdFe nanostructure decorated graphdiyne nanosheet (PdFe/GDY) as a peroxidase mimic because Pd when alloyed with Fe or GDY was previously shown to have the optimum adsorption energy required for accelerated H_2_O_2_ decomposition and stability of oxygen-containing radicals, whereas GDY could act as a promising support due to bonding between its π electron pair and empty d-orbitals of Pd atoms. PdFe/GDY showed surprisingly high peroxidase activity compared to HRP. This was owing to more available adsorption sites on PdFe/GDY for TMB and H_2_O_2_ and low adsorption energy (-0.62 eV) of •OH on the Pd, leading to an increase in H_2_O_2_ decomposition (Wang et al., 2021).

Recently, the effect of doping transition metals (Zn, Ni, and Co) on the catalytic performance of Fe_3_O_4_ nanozymes was evaluated (Vetr et al., 2018), and CoFe_2_O_4_ NPs showed the highest catalytic activity. Later, Wang et al. showed that Co-doped Fe_3_O_4_ nanozymes not only possessed high peroxidase activity but had a 100-fold higher affinity for H_2_O_2_ than Fe_3_O_4_ nanozymes that could catalyze ultralow concentrations (10 nM) of H_2_O_2_. Though Co possesses a similar size as Fe atoms, its higher redox potential Co^3+/^Co^2+^ (1.30 V) than that of Fe^3+/^Fe^2+^ (0.771 V) and ability to produce more catalytically active sites and substrate-binding sites on nanozyme could be responsible for the high peroxidase activity of Co@Fe_3_O_4_ nanozyme (Wang Y. et al., 2019).

Similarly, Jiang et al. have synthesized B-doped core-shell Fe@BC nanozymes, where the Fe core was covered by over ten layers of B-doped carbon shells. The DFT calculations proved that the high peroxidase activity of the nanozyme was attributed to B doping in BCO_2_ that provides more electrons to H_2_O_2_ to promote O–O bond breakdown and a low-energy barrier (-1.16ev) for H_2_O_2_ conversion (Jiang et al., 2021). N- doping also contributed to enhancing the peroxidase activity of Fe_3_C/N-doped graphitic carbon nanomaterials (Fe_3_C/NeC**)** (Li Y. et al., 2020). Nanocomposites made of wide band-gap materials (ZnO) or those possessing crystal defects such as a large number of valence band holes or conduction band electrons possess the capability of generating effective ROS in the cells (Liang et al., 2019).

### 4.2 Nanozyme Surface and Morphology

The catalytic action of nanozymes is affected by a number of factors inherent to the nanozyme such as size, shape, and surface charge, some of which have been explained in recent reviews (Navyatha et al., 2021). The ROS generating capability of nanozymes is strongly affected by the oxidation or valence state of the atom, structural defects, surface roughness, etc. These factors become important in designing a composite nanozyme when its ROS generating capability is intended to be of therapeutic use. Because these properties of nanozymes could affect the bacteria capturing ability of the nanozyme, they generate surface-stabilized ROS, which helps in overcoming the short life span and slow diffusion rate of ROS. It further could make the nanozymes efficiently catalyze much lower and safer H_2_O_2_ concentrations. Defect engineering means modifying the band edges and band-gap energies which increases the catalytic performance of the nanozyme (Twilton et al., 2017), and the rugged nano-morphology provides a trap for bacterial adsorption. Inspired by this, Wang et al. synthesized a defect-rich adhesive MoS_2_/rGO vertical heterostructure as a multienzyme antibacterial mimic (Figure 3A) (Wang et al., 2020). The microwave-assisted hydrothermal method introduced many surface defects with double vacancies for S and Mo. At acidic pH, the nanozyme possessed POD-, CAT-, and OXD-like activities, and the strong ROS species could show excellent antibacterial effects *in situ.* Recent experimental approaches show that oxygen vacancy engineering can create trapping sites for electrons and can be used to improve the catalytic activity of nanomaterials (Huang et al., 2019). Such sites can increase electron-hole recombinations, resulting in the enhanced photothermal performance of nanomaterials (Wang L. et al., 2019a; Wang et al., 2020). Peng et al. reported the first-time use of oxygen vacancy engineering in simultaneously enhancing ROS production and photothermal performance of the MnO_2_@Au nanostructure. The bonding of Au atoms to (O) atoms of MnO_2_ creates an oxygen vacancy in the MnO_2_ lattice. The nanozyme produced highly toxic superoxide radical (•O_2_^−^) and photothermal activity along with GSH depletion capacity, allowing photothermal/oxidation synergistic theranostics TME-regulation (Peng et al., 2020).

### 4.3 Multicatalytic Action

Recently, composite nanozymes possessing multiple catalytic actions, including pro-oxidative and antioxidative functions, have been synthesized. However, their catalytic activities can be tuned by optimizing their pH of action. Some pro-oxidative nanozymes can possess both OXD- and POD-like activity and could act as superior nanozymes by generating both •OH and •O_2_^−^ radicals. For instance, Fe and N co-doping in hollow carbon spheres endowed them with appreciable POD-, CAT-, and SOD-like activities (Fan et al., 2020) POD-like activity generates •OH radicals under weak acidic conditions and is effectively used for bacteria-infected wound healing, whereas CAT/SOD activity scavenged generated H_2_O_2_ and •O_2_ under near neutral conditions and used for noninfectious inflammatory bowel disease. Similarly, pH-switchable Co(OH)_2_/FeOOH/WO_3_ nanoflowers displayed POD activity at acidic pH (<7) and CAT activity at basic pH (>7) and were used for PDT-integrated tumor therapy (Alizadeh et al., 2021).

Nanozymes are also used as multienzyme nanoreactors which display cascade catalytic reactions and are used in tumor therapy (Liu et al., 2020). For instance, GOx activity was integrated with the ROS generating ability of nanozymes and was used for ROS-mediated tumor cell killing along with inducing starvation conditions in the cells. IrRu-GOx@PEG NPs were loaded with natural GOx, which converted tumor-sensitive glucose to H_2_O_2_ and killed tumor cells due to starvation by depleting the nutrient source. Second, IrRu NPs catalyzed endogenously produced H_2_O_2_ to highly toxic singlet oxygen ^1^O_2_ (that mediates oxidative damage), and O_2_ released oxygen helped in continuing the reactions of starvation therapy (Figure 3B) (Wei et al., 2020). Another such cascade nanoreactor, Pd@Pt-GOx/HA, was modified with an outer layer of hyaluronic acid, which could block various catalytic activities of the nanozyme, reduce its cytotoxicity to normal cells, and specifically target CD44-overexpressed tumors (Ming et al., 2020). The HA layer gets decomposed by hyaluronidase in tumor cells and exposes the nanozyme’s catalytic sites. Ultrasmall trimetallic (Pd, Cu, and Fe) alloy nanozyme (PCF-a NEs) displayed glutathione oxidase and POD cascade reactions in circumneutral pH, which was used for tumor CDT in combination with photothermal ablation and ultrasound (Jana et al., 2021).

### 4.4 Decorating With Unique Biomolecules

Introducing surface coatings, chelating ions, or antibiotics on the surface can increase the stability and enzyme mimetic activity of nanostructures. The use of tannic acid for reducing Au (III) to form AuNPs and subsequent chelation with Cu^2+^ ions was used to fabricate a shell-coated Au@TACu nanozyme with increased peroxidase activity and excellent photothermal performance (Liu et al., 2020). Apart from the dual ROS and PTT effects, the acidic TME caused the dissolution of the TACu shell and the released Cu^2+^ depleted the overexpressed GSH, augmenting the oxidative stress–mediated killing of tumor cells. It is well known that CXCR4/CXCL12 interaction leads to acute myeloid leukemia (AML) after chemotherapy and hence the strategy of conjugating the CXCR4 antagonist on the Fe_3_O_4_@Pt composite nanozyme’s surface interferes with this axis (Figure 3C) (Kong et al., 2021). This approach, along with the synergistic action of ROS generated by nanozymes, caused AML cell apoptosis and prevented their migration from bone marrow to other tissues, which in turn prolonged AML mouse survival.

### 4.5 Introducing Multifunctionality

An important aspect of composite nanozyme designing also encompasses endowing them with some additional features in addition to ROS generating ability to enhance their usability and efficacy. For instance, Mu et al. reported reusable nanozymes by synthesizing a super-paramagnetic NiCo_2_O_4_-Au composite that can be easily separated from the media with a magnet. Their antibacterial activity remained intact for three cycles of separation (Mu et al., 2021). MnOx-PLGA@PDA nanoparticles (PP-MnOx) were synthesized as a multifunctional antitumor platform by integrating the oxidase mimic potential of MnOx and its ability to act as a sensitive T1-weighted magnetic resonance imaging agent with the cytotoxic effect of artesunate encapsulated within the PLGA core (Xi et al., 2021). The multifunctionality feature of composite nanozymes can also be augmented by combining their ROS generating ability with PTT and PDT. The synergistic actions of these modalities have shown accelerated antitumor and antibacterial effects. For effective CDT, nanozymes catalyzing the oxidation of intracellular glutathione were also designed.

Light has been shown to activate the enzyme-like properties of nanozymes (Liu et al., 2019). Liu et al. have studied the effect of surface plasmon resonance (SPR) on activating the POD-like activity of CeO_2_ for antibacterial action. Au@CeO_2_ nanozymes were synthesized with low oxidase and peroxidase-like activity, which was enhanced three times after 808-nm laser irradiation as compared to only CeO_2_ NPs or Au nanorods at weekly acidic pH (Liu et al., 2021). Xu et al. synthesized a multifunctional platform enabling photothermal/chemodynamic/pharmaco-synergistic antibacterial action by encapsulating tungsten sulfide quantum dot (WS_2_QD) nanozyme and vancomycin antibiotic in a thermal-sensitive liposome. POD mimic activity of WS_2_QD generated radicals; OXD mimic activity catalyzed GSH oxidation, causing its depletion; and thermal sensitivity improved catalytic activity and liposome rupture, facilitating targeted drug delivery (Figure 3D) (Xu et al., 2020).

Thus, the first strategy for designing efficient ROS-generating composite nanozymes should be to judiciously choose constituent elements that can enhance electron transfer to generate ROS, provide low adsorption energy to stabilize the generated radicals on the nanozymes surface, possess higher redox potentials and high affinity, could act as support for POD mimic elements, or could allow plasmon-induced electron transfer to other constituent elements. These properties, besides being intrinsic characteristics of an element, can also be improved to some extent by modifications in synthesis conditions (reactants and stabilizers used, method of synthesis, pH, temperature of synthesis, calcinations etc.) of the composite nanozymes. Modifications in synthesis conditions can directly influence their surface topography and morphology, for example, creating surface defects, roughness, oxygen vacancies, electron-hole pairs, making them porous, or having pseudopodia-like extensions can directly impact the intrinsic catalytic behavior of the composite nanozymes. Since catalysis is a surface phenomenon, such modifications can have nanozymes with improved Km and Vmax values, which can be used to catalyze low H_2_O_2_ concentrations or *in situ* produced H_2_O_2_. In addition, the overall bacterial/tumor cell killing ability of composite nanozymes can be accelerated by exploring multienzyme mimic synergistic activities within a nanozyme and prohibiting the antagonistic catalytic activity. For instance, heteroatom doping in carbon nanomaterials confers multimimic activity besides enhancing the enzymatic efficiency. Making cascade nanozymes and integration with PTT or PDT can also enhance their specific cell killing ability.

## 5 Research Progress in ROS-Mediated Therapeutic Applications of Composite Nanozymes

Composite nanozymes designed recently show both intrinsic peroxidase- and oxidase-like activity to generate ROS •OH, •O_2_^−^, and H_2_O_2,_ which can vividly damage more lipids, amino acids, and polysaccharides in the bacterial membrane and also kill cancerous cells due to their oxidative and electrophilic nature (Li D. et al., 2020). Hence, the pro-oxidative potential of these nanozymes has been used for diverse therapeutic applications, as discussed ahead.

### 5.1 Antibacterial and Wound Healing Applications

**FIGURE 4:**
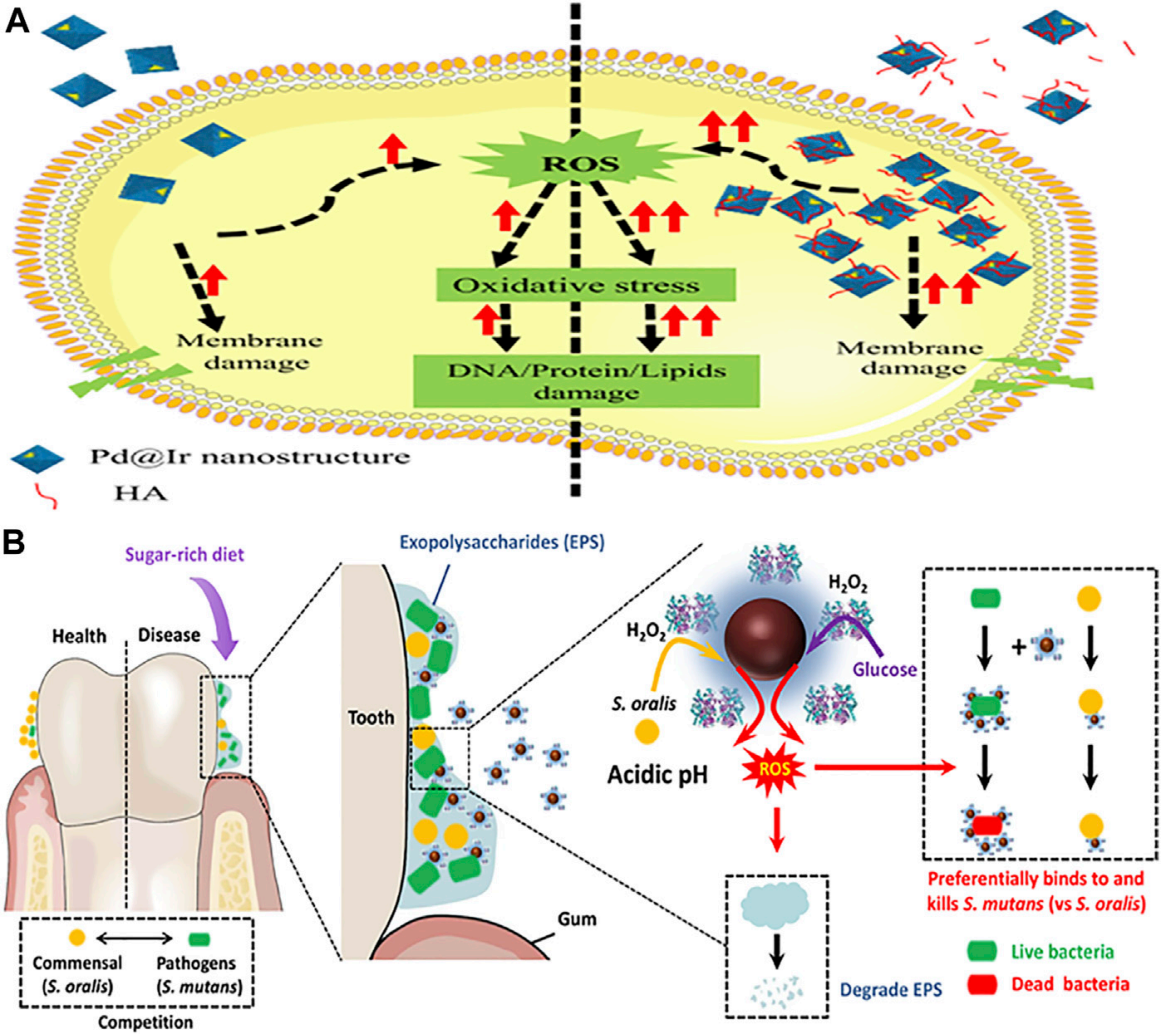
Schematic representation of composite nanozymes as effective antibacterial and antibiofilm agents. **(A)**. Mechanism of enzyme mimic Pd@Ir nanostructures for potential antibacterial therapy. Adapted and reprinted with permission from the reference (Cai et al., 2019). HA: humic acid. **(B)**. Dual-catalytic activity of Dex-IONP-GOx for the disruption and inhibition of the bacterial biofilm. Adapted and reprinted with permission from the reference (Huang et al., 2021).

Composite nanozymes have shown broad-spectrum nanoantibiotic effects by killing gram-positive, gram-negative, and multidrug resistant bacteria at low and biologically safe H_2_O_2_ concentrations (Chen et al., 2020). Cai et al. designed POD and OXD mimics of Pd@Ir nanohybrid which killed bacteria by causing oxidative stress **(** Figure 4A **)** (Cai et al., 2019). Composite formation of CuS with graphene oxide was carried out to synthesize a needle-shaped nanozyme that could puncture bacterial membranes in addition to toxic •OH generation through its POD and OXD mimic activity (Wang et al., 2020). It could effectively eliminate MRSA and accelerate MRSA-infected wound healing. Nanostructures are also being explored as efficient photocatalysts for antibacterial photocatalytic therapy. For instance, an all-organic heterostructure g-C_3_N_4_ nanosheet loaded with self-assembled perylene-3,4,9,10-tetracarboxylic diimide (PDINH) was synthesized and shown to generate active species such as •O_2_^−^, ^1^O_2_, and •OH upon visible-IR light irradiation (Wang L. et al., 2019b). The active radicals diffused and efficiently killed both gram-positive and gram-negative bacteria. The photocatalyst accelerated the healing of bacteria-infected skin and promoted collagen regeneration. Mixed-metal oxides (Co-Al-Ce) prepared by compounding CeO_2_ with Co_3_O_4_ and CoAl_2_O_4_ showed maximum POD activity at 2.5% Ce and 200°C calcination temperature (Chen et al., 2020). It exhibited maximum antibacterial activity against Gram-negative bacteria at near-neutral pH. For efficient wound healing, it is important to regulate infection-related host responses besides eradicating bacteria from the wound site. Hemin@Phmg-TA-MSN was demonstrated as a dual-functional nanomedicine (Wei Z. et al., 2021). Graphdiyne-hemin (GDY-hemin) composite was synthesized as a stable nanozyme displaying bacterial cell killing at the wound site and accelerated wound healing mediated by ROS produced by its POD activity (Ali et al., 2021). The UiO-66-NH_2_/CS composite membrane exhibited POD activity and could generate active oxygen (•OH) before UV irradiation at a trace amount of H_2_O_2_. With UV irradiation, Zr^4+^ changes to Zr^3+,^ and after removal of UV light, Zr^3+^ loses electrons to form Zr^4+,^ which was used to produce H_2_O_2_ and •O_2_^−^. The membrane exhibits high antibacterial activity and has potential in wound dressings (Wang et al., 2021). Table 3 enlists composite nanozymes with promising potential as antibacterial or antibiofilm agents.

**TABLE 3:** Various composite nanozymes with demonstrated use as antibacterial and wound healing agent through ROS.

S. no.	Nanozyme used	Structural property of nanozymes leading to catalytic action	Catalytic property shown	Working pH of nanozymes	Km	Vmax	ROS species generated	Demonstrated application	Mechanism of action	Other specific property of nanozymes	Ref
1	CuCo_2_S_4_	Uniform, well-crystallized cubic spinel, size -30 nm	POD	7.4	209.9 mM	232.8 n M·s ^−1^	•OH	AB, WH	Oxidative stress	Antibiofilm	Li et al. (2020)
2	Dealloyed porous Pt/Ag nanoparticles	Porous, Pt-enriched octahedron, size range - 20.9–22.1 nm	POD, OXD, CAT-	4.0	0.86 mM	34.75 (×10–8 M s^−1^)	•OH	AB	Oxidative stress, disrupts permeability of the membrane	As biosensors, and biomedicine	Cai et al. (2017)
3	Cu_2_WS_4_ nanocrystals	Cuboid, size ∼20 nm	POD	4	—	—	H_2_O_2_ and •OH	AB, WH	oxidative stress	Selective and good bacteria-binding ability	Shan et al. (2019)
4	Cu_2_MoS_4_	Uniform morphology, size is ≈ 28 nm	POD, OXD	4	OXD-12.06 µm, POD- 25.46	OXD- 0.11 μm s^−1^	H_2_O_2_, •OH	AB	Oxidative stress	Enhanced activity under NIR-II	Shan et al. (2020)
	OXD-42.81 × 10^–8^										
5	(MoS_2_)/rGO	Defect-rich surface	POD, OXD	3.0	POD-0.26 mM	POD**-**25.6 (10^–8^ M/s)	•OH	AB,WH	Structural deformation. Causes oxidative stress through GSH (antioxidant) consumption and ROS generation	Defect and light irradiated improved activity, Also, GSH consumption ability	Wang et al. (2020)
6	CuS/GO	Needle-like	POD, OXD	4.6	—	—	•OH	AB, WH	Nanoknife mechanism (puncture bacterial membranes), and though generation of ROS	Good biocompatibility	Wang et al. (2020)
7	UsAuNPs/MOFs	2D ultrathin morphology	POD	pH 5.0	7.94 × 10^–3^ m	-	•OH	AB,WH	Oxidative stress	Negligible biological toxicity	Hu et al. (2020)
8	Fe/N-HCNs	Hollow porous	POD, OXD	3.5	—	—	•OH	WH	ROS destroyed bacterial cells treat bacteria-infected inflammation	CAT and SOD activity treat noninfectious inflammation	Fan et al. (2020)
9	Pd@Ir	Octahedral core shell structured, size ∼14 nm	POD, OXD	4.0	0.28 mM	0.079 (10–^7^ Ms^−1)^	H_2_O_2,_ •OH	AB	Oxidative stress, damage membrane	Biocompatible	Cai et al. (2019)
10	Au@CeO_2_	Uniform dumbbell-shape size ∼20 nm	POD	3.0	0.006 mM	13.34 nM S^−1^	OH and ^1^O_2_	AB	Oxidative stress	Good catalytic stability and durability	Liu et al. (2021)
11	NSP-CQDs	Spherical nature and excellent dispersibility, size 2–6 nm	POD	4.0	32.61 mM	6,950.68 10^−8^ Ms^−1^	•OH	AB	•OH, attacking the bacterial cell membrane	Applicable in immunoassays, biotechnology, and clinical diagnosis	Tripathi et al. (2020)
12	L-Arg/GOx@CuBDC	Sea urchin–like, Size-250 ± 50 nm	GOx, POD, NOS	7.4	—	—	•OH, •O_2_	AB	ROS and RNS oxidize and degrade organics, including penetrating cell membrane, reacting with biological substrates (lipids, proteins, DNA, and RNA)	High specificity	Cheng et al. (2020)
13	Co-Al-Ce mixed metal oxide	Good dispersion of catalytically active components and high specific surface area	POD	4	32.9 mmol/L	—	•O_2_	AB	Oxidative stress	Application in marine antifouling	Chan et al. (2020)
14	Nickel disulfide	Monodispersed and uniform spherical, porous, diameter -112.31 nm	POD	—	∼3.64 mM	∼1.55 × 10̵^4^ mM min̵^1^	•OH	AB	Cell wall damage by ROS, consume GSH in bacteria	Photothermal activity	Wang et al. (2020)
15	MoS_2_-Hydrogel	Positively charged porous, Flower like diameter- 165 nm	POD	4	—	—	• OH	AB	Damage membrane and causes oxidative stress	Photothermal properties under visible and NIR region	Sang et al. (2019)
16	Hydrogel-based artificial enzyme (copper and L-aspartic acid)	Network nanofiber diameters -50–70 nm	POD	7.4	38 Mm	9.6 × 10^−8^M S^−1^	• OH	WH, AB	ROS Oxidize cell membrane of bacteria	Negligible toxicity and high biocompatibility	Qiu et al. (2020)
17	WS_2_QDs-Van@ lipo	Spherical uniform size less than 10 nm	POD, OXD	2–4	—	—	• OH	AB and antibiofilm	ROS and drug mediated	Oxidize GSH improve CDT PTT/pharmaco synergistic antibacterial therapy, NIR-controlled drug release	Xu et al. (2020)
18	GQD/AgNP hybrids	Size-2–10 nm	OXD, POD	5–7	—	—	OH,•O_2_^−^	AB	ROS-mediated oxidative stress and disruption of bacterial membrane	Photothermal activity	Chen et al. (2017)
19	CaO_2_/H-G@alginate	2D nanosheet	POD	5	2.568 mM	0.185 μM S^−1^	hROS	Antibiofilm	hROS can damage the main component of biofilm	No need of H_2_O_2_	Yan et al. (2018)
20	Au/g-C_3_N_4_	Size- 150 nm	POD	5.0–7.4	60.0 ± 3.21 (10^−5^ M)	150.8 ± 4.95 (10^−7^M·S^−1)^	•OH	AB, WH	ROS-mediated oxidative stress	Antibiofilm	Wang et al. (2017)
21	2D MOF (2D Cu-TCPP(Fe) GOx (MOF (2D Cu-TCPP(Fe)/GOx	Sheet-like structure, Crystal size 13.6	POD	3–4	—	—	•OH	AB, WH	•OH-induced oxidative damage	GOx convert glucose into abundant gluconic acid and H_2_O_2_ avoiding the use of toxic H_2_O_2_. negligible biotoxicity	Liu et al. (2019)
22	MSN-Au NPs	Bean-like size-500 nm	POD, OXD	4	15.81 ± 0.76 mM	12.66 ± 0.36 (10-8M^·^ s ^−1^)	•OH, •O_2_−^1^O_2_	AB	ROS-induced oxidative stress	Antibiofilm	Tao and Ju, (2015)
23	PdFe/GDY	Wrinkled nanosheet, size-	POD	4	0.1653 mM	0.9711 10^–8^ M s^−1^	•OH	AB,WH	ROS-mediating bacterial cell membrane destruction	GSH activity	Wang et al. (2021)
24	PEG-MoS_2_ NFs	Flower-like, diameter-25 nm	POD	3–4	2.812 mmol L^−1^	3.88 × 10^−7^	•OH	AB, wound healing	ROS and hyperthermia-mediated oxidative damage	PTT and accelerated GSH oxidation in the 808-nm laser	Yin et al. (2016)
25	Co_4_S_3_/Co(OH)_2_	Tube-like diameter -∼70 nm	OXD	4	1.33 mM	4.66 7–10 M/s	O^2−^, ^1^O_2_	AB	ROS-induced oxidative damage	Complete sterilization without H_2_O_2_	Hu et al. (2018)
26	Cu-HCSs/H_2_O_2_	Spherical and hollow structure diameter -∼100 nm	POD	4.5	—	—	•OH	AB	ROS and released Cu^2+^ caused membrane damage, lipid peroxidation, and DNA degradation of bacteria	Used to treat intestine infection induced by S. typhimurium	Xi et al. (2019)
27	NiCo_2_O_4_-Au	Tube- like rough surface	POD, OXD	4	28.33 ± 7.304 (10^–3^ mM)	28.773 ± 0.103 (M/S)	•OH, •O^2−^, ^1^O_2_	AB, WH	ROS-induced oxidative damage	Antibiofilm, recyclable	Mu et al. (2021)
28	Co-V MMO Nanowires	Nanowire with rich surface defects	POD, OXD	4	0.12 (mM)	5.3 (10^–8^ M/s)	•O_2_, •OH	AB	ROS-induced oxidative damage	Application in the fields of new energy and catalysis	Wang et al. (2020)
29	Co_4_S_3_/CO_3_O_4_	Hollow tube-like diameter -∼166.7 nm	POD, OXD	4	0.17/mM	1.6 × 10^−5^/M/s	OH•, ^1^O_2_, •O_2_^−^	AB	ROS-mediated oxidative stress	No need of H_2_O_2_, good selectivity, promising recyclability, and reliable	Wang et al. (2020)
30	Fe_3_O_4_@MoS_2_-Ag	Defect-rich rough surface, diameter ∼428.9 nm	POD	4	1.00 (mmol/L)	1.11 (✕10^−7^mol/(L·s)	OH•	AB	Toxic ·OH and Ag + assisted by local hyperthermia attack the bacterial membranes	Adhesive ability Reusable	Wei et al. (2021)
31	Ir−Ag−IrO_2_	Uniform and rough surface, size -90 nm	POD	3	67.94 ± 3.83 μM s−1	0.3193 ± 0.0517 M	• OH	AB	ROS-mediated oxidative stress	More precise and selective local treatment	Yim et al. (2020)

### 5.2 Anti-Biofilm

One of the strategies to prevent biofilm formation on surfaces is to incorporate antibacterial and anti-adhesive coatings on these surfaces. In this context, the strategy of using nanomaterials for designing super hydrophobic (Ren et al., 2018), and more stable hydrophilic/superhydrophilic surfaces (Valencia et al., 2018) was explored. But these coatings were susceptible to damage from complex environmental changes. To address this issue, the recent approach is to design smart responsive materials as anti-adhesive materials and possessing the switchable function of being antibacterial (Ghasemlou et al., 2019). Very recently, Lin et al. designed a light and thermoresponsive adjustable anti-adhesion surface using light-responsive titanium oxide nanotubes as a base. Thermo-responsive P (vinylcaprolactam (VCL)–co-polyethylene glycol-methacrylate (PEGMA)–co-alkyl-dimethyl tertiary amine (QAS)–co-vinyltrimethoxysilane (VTMO)) copolymer was grafted on TiO_2_ surface along with antibacterial QAS constituent and anti-adhesive PEGMA polymer (Lin et al., 2021). Hence, due to VCL-triggered conformational change, bacterial antiadhesion was enhanced by thermoresponse.

*Streptococcus mutans* thrives well in sugar-rich and acidic microenvironments associated with dental caries. The antagonist action of commensals such as *S. oralis* is limited due to low concentrations of intrinsic H_2_O_2_ production and its confined effect. Hence, a bifunctional nanohybrid system possessing dual-catalytic activity was designed by conjugating GOx on dextran-coated iron oxide NPs (Figure 4B) (Huang et al., 2021). GOx catalyzed abundant glucose available in dental caries to H_2_O_2_ and dextran coated iron oxide NPs converted it to generate ROS that killed bacteria and degraded the EPS matrix. The nanohybrid system acted as a targeted antibiofilm agent *in vivo* compared to the gold standard drug chlorhexidine.

### 5.3 Antitumor

**FIGURE 5:**
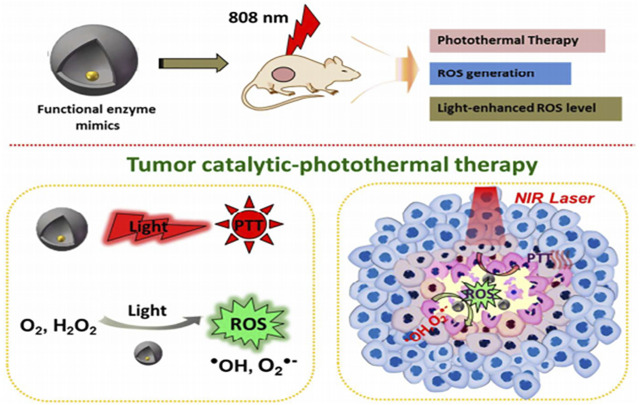
Schematic of representation of yolk-shell gold@carbon nanozymes with intrinsic enzyme mimic activity and photothermal response as an effective antitumor agent Adapted and reprinted with permission from (Fan, 2018).

Nanozymes display strong ROS-mediated antitumor potential, and considerable endeavors are made to realize this potential. However, the complex TME limits their therapeutic efficacy and drives the need for a TME-specific and endogenously responsive approach for efficiently utilizing nanocatalytic therapy. The TME is characterized by a weakly acidic environment, high glutathione (GSH) concentrations, overproduction of H_2_O_2_ (50–100 × 10^−6^ m) hypoxia, and an immunosuppressive environment. High GSH concentrations could offshoot the pro-oxidative nanocatalytic effect of nanozymes (Dong et al., 2020). To realize this, Fan et al., developed yolk-shell gold@carbon nanozymes with a synergistic action of photothermal-enhanced catalytic activity (Figure 5) (Fan L. et al., 2018). An et al., synthesized MnO_2_@HMCu_2_–xS nanocomposite with photothermal-enhanced GSH depletion ability exhibiting tumor destruction (An et al., 2019). Dong et al. have integrated hyperthermia-enhanced dual-enzyme mimic activity (POD and CAT mimic) with GSH depletion capability in cerium-based nanozyme (PEG/Ce-Bi@DMSN) for improved tumor ablation (Dong et al., 2020). Fe-based NPs show Fenton reaction for •OH radical generation, but the reduction of Fe^3+^ by H_2_O_2_ is hampered due to weakly acidic TME. The pH dependency of nanozyme was attempted to be overcome by using NIR energy (Nie et al., 2019) and co-delivery of nanocatalysts and iron species (Zhao P. et al., 2019) to drive Fe^3+^ reduction; however, electron delivery between Fe^2+^ and Fe^3+^ was not efficient at mild acidic pH. The hurdle was overcome recently when Fe, Al, and N co-incorporated graphitic nanozyme (Fe/Al-GNE) were synthesized to boost electron transfer capacity (Li J. et al., 2020), Al functioned as an electron pump to transfer electrons to Fe^3+^ and the graphitic sheet accelerated the process of electron delivery. Thus, Fe/Al-GNE nanozymes could efficiently catalyze excess H_2_O_2_ in the TME, inducing apoptosis of tumor cells. Yang et al. constructed bimetallic NPs on a metal organic framework to be used as hosts instead of Fenton agents. Among them, Cu-Pd@MIL-101 exhibited excellent POD-like activity along with GSH depletion ability, which synergistically favored CDT for tumors. Hypoxic TME favors tumor growth, which could be minimized by constant oxygen supply (Yang P. et al., 2021c). To this end, Ru@MnO_2_ nanozymes were coated with erythrocyte membrane that increases its biocompatibility and circulation time in blood (Zhu et al., 2021). These nanozymes catalyzed endogenous H_2_O_2_ to generate O_2_ to relieve hypoxia and support PTT and CDT. With an intention to overcome immunosuppressive TME, TGF-β inhibitor (TI) was loaded into PEGylated iron manganese silicate nanoparticles (MSN-PEG-TI) (Xu et al., 2020). Both IMSN and TI promoted polarization of macrophages from M2 to M1 and induced H_2_O_2_ regeneration, which accelerated the POD-like activities of IMSN nanozyme. This immunomodulation-enhanced nanozyme activity showed a potent antitumor effect on multicellular tumor spheroids (MCTS) and *in vivo* CT26-tumor-bearing mouse models. A sufficient level of endogenous H_2_O_2_ was needed to produce •OH radicals for efficient tumor therapy, and it was achieved by developing nanoplatforms with cascade catalytic abilities as described in Section 4.3. However, the extremely short half-life (∼1 µs) of •OH restricts its future application. This problem was addressed by constructing Fe_3_O_4_ nanocrystals coated with Schwertmannite (Sch) matrix (Sch@Fe_3_O_4_), which possessed the ability to generate SO^4^•^-^ radicals from SO_4_^2-^ when attacked by •OH radicals in a cascade reaction (Wu et al., 2021). Fe_3_O_4_ hollow core generates •OH in Fenton’s reaction, which is converted to SO_4_•- (half-life of ∼30 µs) by nanoscale cellular pseudopodia-shaped Sch grown on the Fe_3_O_4_ core. The combined effect of two radicals, along with PTT and GSH depletion mediated by L-buthionine sulfoximine (BSO) molecules loaded in the hollow Fe_3_O_4_ cores, showed efficient tumor cell death.

The antitumor potential of nanozymes is also realized by integrating MnOx nanozymes with artesunate-loaded drug carrier Poly-(lactic-glycolic acid) PLGA to form PLGA@PDA nanoparticles (PP-MnOx NPs) (Xi et al., 2021). PDA was used to link nanozyme with PLGA. The nanozyme exhibited OXD-mimicking activity to catalyze the conversion of O_2_ and generate ROS. Mechanistic insight showed that the electrons released during conversion of Mn^2+/^Mn^3+^ on the surface of nanozyme to Mn^4+^ were trapped by O_2_ to form H_2_O_2_. Activation of mitochondrial apoptotic pathways due to synergistic action of ROS and sustained release of the drug artesunate leads to efficient tumor cell death. The ROS generating capability of Fe_3_(PO_4_)_2_ 8H_2_O–CDs (carbon nanodots)–FA hybrid nanoflowers (hNFs) was found to effectively kill cancerous cell lines in the presence of exogenous H_2_O_2_ and in combination with ascorbic acid mediated endogenously produced H_2_O_2_ (Guo et al., 2019). In another approach, the ROS generating capability of Au–Ag@HA NPs was combined with a radiation paradigm for effectively killing tumor cells (Chong et al., 2020). Ionizing radiation boosted •OH radical production and Ag^+^ release at the tumor site, thereby synergistically killing 4T1 breast cancer cells. Urchin-like Fe–MIL-88B–NH_2_@PFC-1-GOx (MPG) nanoparticles were constructed as versatile nanoplatform to synergistically offer CDT, sonodynamic therapy, and starvation therapy to effectively kill tumor cells (Hu et al., 2021). Table 4 enlists such composite nanozymes with demonstrated anticancerous potential mediated through ROS.

**TABLE 4:** List of composite nanozymes with demonstrated use as an antitumor agent through ROS.

S. no.	Nanozyme used	Structural property of nanozyme leading to catalytic action	Catalytic property shown	Working pH of nanozyme	Km	Vmax	ROS species generate	Mechanism of action	Other specific property of nanozyme	Ref
1	Fe_3_O_4_@Bi_2_S_3_	Virus-like diameter∼ 80 nm	POD	5.5	—	—	• OH	ROS kill cancer cells	Possesses photoacoustic and (PA) Infrared Thermal Imaging (IRT) imaging capacity. Photothermal activity under near-infrared (NIR), recyclable	Zhao et al. (2020)
2	AgPd@BSA/DOX	Rough core-shell branched structure, Size ∼120 nm	POD	5.5	—	—	• OH	Through hyperthermia with the photoreactive release of chemotherapy drug and ROS generation	Have photothermal conversion ability under NIR laser irradiation. A nanocarrier of drug doxorubicin	Li et al. (2020)
3	DMSN-Au-Fe_3_O_4_	Central-radial pore structures, diameter ∼140 nm	POD, GOx	6.5	10.10 × 10^–3^ m	1.996 × 10^–8^ m s^−1^	H_2_O_2,_• OH	ROS-induced apoptosis of cancerous cells	Excellent biosafety, easy excretion	Gao et al. (2019)
4	CD44MMSN/AuNPs	Wrinkle structure, diameter ∼50 nm	POD, OXD	3.6	6.35 mM	3.85 × 10^–8^ Ms^−1^	H_2_O_2,_•OH	ROS-mediated apoptosis	Tumor-specific precision therapy, “toxic-drug-free” and non-invasive nanocatalytic biomedicine	Wang et al. (2020)
5	Au@HCNs	Spheres, diameter- 180 nm	OXD, POD	4.5	0XD-0.170 mM POD-0.0323 Mm	OXD-4.92 × 10^−8^ POD-33.00	,•OH	light-enhanced ROS generation and photothermal-induced killing of cancerous cells	Excellent NIR absorbing agents for tumor PTT	Fan et al. (2018)
6	PEG-Cu_2_Se HNCs a	Hollow nanocube diameter- 86.89 ± 19.93 nm	POD	7	—	—	• OH	ROS and photothermal-mediated	Good PCE under NIR II window	Wang X. et al. (2019c)
7	Fe_3_O_4_@Pt@ E5	Janus structure Size-(Fe_3_O_4_ 8.0 ± 1.0 nm and Pt 2.0 ± 0.5 nm)	POD, OXD	4.0	22.17 mM	0.139 µMs^-1^	•OH	Apoptosis by generation of ROS and block the CXCR4/CXCL12 axis	Negligible side effects	Kong et al. (2021)
8	MIL-101@BSA-AuNCs NPs	Possessed good dispersibility, size -150 nm	POD	7.4	—	—	·OH	By generation of ROS	Act as thermal sensitization agents under microwave radiation, possesses dual modality imaging property	Ma et al. (2019)
9	NMIL-100@GOx@C	Polyhedral shape, size- 175 nm	OXD	—	—	—	H_2_O_2_	ferroptosis and starvation treatment	Perform Fenton reaction and catalyze H_2_O_2_ (oxidase convert glucose n to gluconic acid and H_2_O_2_) produce ·OH	Wan et al. (2020)
10	GOx@Pd@ZIF-8	Irregular sphere, size-130 nm	POD	—	—	—	·OH	By ROS-mediated apoptosis and glucose starvation	Inhibiting proliferation of cancerous cell both *in vivo* and *in vitro*	Jin et al. (2020)
11	CPGL (GOD, LOD, and C-dots were loaded into the hydrophobic core with the aid of PLGA.)	uniformly distributed, and granular diameters- ∼ 7–12 nm	POD	4.55	0.02827 ± 0.00873 mM	36.3782 ± 5.24999 mM s^−1^	·OH	By glucose starvation and ROS generation	pH-sensitive ability, excellent tumor-homing ability with good biocompatibility	Wang et al. (2020)
12	PtFe@Fe_3_O_4_	—	POD, CAT	2.5–6.5	53.55 mM	1.078 × 10^−7^	•OH, O_2_ •^ –^	Through ROS generation	Bimodal contrast agent for computed tomography (CT) and PA imaging, -guided diagnosis, Exhibit photo-enhanced catalytic activities under NIR	Li et al. (2019)
13	Fe_3_O_4_@PPy@GOD NCs	uniform with an ∼163.5 nm	POD	6.5	1.59 mm	2.64 × 10^–9^ M s^−1^	•OH	By glucose starvation and ROS-mediated apoptosis	dual-modality diagnostic imaging-guided synergistic nanocatalytic cancer therapy and photothermal-triggered cancer hyperthermia. efficiency in NIR-I and NIR-II biowindows	Feng et al. (2019)
14	N-PCNs	Porous nanospheres, size 100 ± 10 nm	POD, OXD	4.5	OXD-0.084 mM, POD-130 mM	OXD-0.42 10^−8^ M s ^−1^, POD-32.5 10^−8^ M s ^−1^	H_2_O_2_ and •OH	By upregulation of ROS	Also possess CAT- and SOD- like activity	Fan et al. (2018)
15	MnO_2_@PtCo	Nanoflowers, size - 3 nm	POD, CAT	2.5–6.8	—	—	•OH production	ROS-mediated apoptosis	CAT activity help overcome hypoxic condition and enhance the catalytic activity of PtCo	Wang et al. (2018)
16	GSF@AuNPs	2D nanostructured, size 100–400 nm	POD	5	5.980 mM	27.7 × 10^–7^ M s^−1^	OH•	Oxidative stress by ROS	Utilized as a selective, quantitative, and fast colorimetric detection probe for cancer cells	Maji et al. (2015)
**17**	Cu_2_-xTe	cuboid structure, size -30 nm	Glutathione oxidase, POD	5	Glutathione oxidase 0.19 ± 0.03 Mm, POD-135 ± 10 Mm	Glutathionoxidase-19.3 ± 1.1 μM s^−1^ POD- 87 ± 0.02	OH•	Intratumoral oxidative stress to induce immunogenic cell death	Consume GSH and exhibit photothermal activity under NIR-II Light	Wen et al. (2019)
18	Fe_3_O_4_@C NPs	Core−shell structure, size -120 nm	POD	3	0.38 mM	73.99 × 10^–8^ M s^−1^	• OH	ROS-mediated oxidative stress	Selectively, magnetic responsiveness and receptor-binding specificity	An et al. (2013)
19	Magnetic hydrogel nanozyme (MHZ)	Spherical core−shell structure, size 30–50 nm	POD	5.2	—	—	• OH	Oxidative stress damages the protective heat shock protein 70	Powerful platform for combination with hyperthermia and catalytic therapy	Wu H. et al. (2019a)
20	HCS@Pt-Ce6	—	POD, OXD	4.5	POD-0.04853 mM, OXD-0.352 mM	POD-21.7871 10^−8^ M⋅s^−1^, OXD-0.8243 10^−8^ M⋅s^−1^	• OH	ROS and photodynamic mediated apoptosis	Synergistic photodynamic-catalytic therapy of tumor	Xu et al. (2020)
21	AuPt@SF (APS)	Intriguing nonregular polyhedral structure, Size ∼36 nm68.71 ± 32.8 nm	GOx, POD.	5.5	POD-28.148 mM, GOx45.795 μg/ml	POD-6.756, GOx -0.125 μM/s	O^2^—and •OH	Through deleterious tumor starvation and irreversible oxidative-stress destruction	GSH depletion	Yang R. et al. (2021b)
22	CoO@AuPt	Hollow, diameter ∼36 nm	OXD, POD	GOx -6.5, POD and CAT-6.8	—	—	O_2_,•OH H_2_O_2_	ROS and glucose starvation–mediated inhibition of tumor	Also possess CAT activity	Fu et al. (2020)
									Deplete Glutathione	
23	UMOFs@ Au NPs	Core shell structure size-29.8 ± 2.2 nm	OXD	4.5	44.27 mM	12.96 × 10^–7^ M s^−1^	H_2_O_2_ and^1^ O_2_	Glucose starvation and ROS mediated	PDT effects under NIR light irradiation	He et al. (2020)
24	PEG/Ce-Bi@DMSN	Bacteria like	POD, CAT	5.5	27.54 × 10^–3^ m	3.85 × 10^–8^ m s^−1^	• OH	impaired the antioxidant defenses of tumor cells and causes oxidative stress	Deplete GSH, and also Act as PTT agent in the NIRII- biowindow	Dong et al. (2020)

## 6 Limitations and Challenges of Composite Nanozymes

As discussed above, composite nanozymes have presented themselves as multifunctional catalytic agents for disease therapy. Despite the numerous advantages offered by composite nanozymes, their translation from the laboratory to field is far from reality because of the limitations and challenges faced by them. The first and foremost limitation is their low selectivity toward the target cell, which could raise concern about their toxicity and off-target effects. Second, composite nanozymes at times display multiple enzyme mimic activities, such as pro-oxidative (peroxidase and oxidase) and antioxidative activity (superoxide dismutase and catalase) at the same time, which could interfere with desired activity in the living system or may cause a reverse effect. Third, the optimal catalytic activity of many composite nanozymes is restricted to acidic pH, which is not compatible with the physiological and biological conditions. The peroxidase activity of nanozymes is also dependent on the use of H_2_O_2,_ which itself could be toxic beyond a threshold. Hence, pro-oxidative nanozymes that do not depend on the use of H_2_O_2_ would be more suitable and welcomed. Another important issue with composite nanozymes is their biosafety. Composite nanozymes intended to be used for biomedical applications are engineered to interact with cells/tissue. However, broad focus remains on the therapeutic performance of composite nanozymes while ignoring their biosafety assessment. For instance, inorganic nanoparticles mimicking enzyme-like activity frequently accumulate in the reticuloendothelial system (RES), resulting in low passive targeting specificity and long-term toxicity, limiting their use in clinical trials (Yang et al., 2019). Some nanozymes such as nickel disulfide showed good biodegradable properties but had relatively long blood circulation times, which limits their practical applications (Wang et al., 2020). The size, composition, surface charge, dose, and functional groups of composite nanozymes affect their kinetics, specificity, and toxicity. For instance, increasing the size of graphene oxide can improve the potential for bacteria killing but poses toxicity to normal cells and tissues (Mei et al., 2021). Metal-based composite nanozymes not only display good therapeutic effects but also impose a potential health issue due to ionic dissolution such as Zn^2+^ and Cu^2+^ that interact with biomolecules such as proteins and enzymes, inactivating them and causing metal poisoning to cells and tissue (Halbus et al., 2017).

Hence, it is equally critical to conduct a systematic assessment of nanozyme biosafety in terms of assessing their absorption, biodistribution, metabolism, clearance mechanism, pharmacokinetics, H_2_O_2_ concentration, and long-term toxicity in addition to their therapeutic effect in *in vivo* studies. Furthermore, long-term toxicity studies involving particle size, shape, and surface chemistry are required to ensure the nanozymes are suitable for *in vivo* biological applications. In addition, research into nano-bio interfaces, nanozyme immunotoxicity, genotoxicity, and neurotoxicity from molecules to organisms is still in progress and has to be thoroughly investigated. Because these nanomaterials are designed to interact with cells, it is critical that these interactions do not have a negative impact on the human body.

## 7 Conclusion and Perspective

In this review, the recent developments in using the intrinsic ROS generating ability of composite nanozymes for various therapeutic applications are presented and understood. Composite nanozymes score over other nanozymes in offering unique inherent properties of constituent elements that synergistically enhance their applicability. For instance, Ni nanoparticles barely oxidize the enzyme substrate, whereas Pd NPs possess multienzyme mimic activity, and NiPd nanoparticles exhibit higher catalytic activity than any of these alone (Wang Q. et al., 2016). The pro-oxidative composite nanozymes have found increased applicability as antibacterial, antibiofilm, and antitumor agents. It is due to their ability to alleviate limitations of existing ROS generating nanozymes such as 1) inefficiency to produce significant levels of ROS to kill bacteria at biologically safe low H_2_O_2_ concentrations; 2) single-modal nanozymes cannot effectively eradicate resistant bacteria or abnormal cells, 3) inefficient capture of H_2_O_2_ or generated radicals on bacterial surface; and 4) limited catalytic activity in the TME.

The composite nanozymes provide flexibility in their design and synthesis by offering a diverse choice of elements to be used for support or as dispersed nanoparticles. Tailoring nanozymes to closely mimic natural enzymes can be used as a strategy to design efficient composite structures. For instance, Zhang et al. have used covalent organic frameworks (COFs) to tailor the pore microenvironment around active centers and enhance the catalytic ability of MOFs (Zhang et al., 2021). The pseudopodia-like structure of the COF enabled the nanozyme platform to capture bacteria efficiently through multivalent interactions between hairy bacteria and spiky COFs. The various strategies discussed for engineering composite nanozymes in this review can be used for accelerating their ROS generating ability, making them multifunctional (photothermal, optical, photodynamic, and chemodynamic activity), adhesive, reusable, and compatible to act at neutral or near-neutral pH and in hypoxic TME, efficient enough to eliminate multidrug resistant bacteria/biofilm and targeted tumor cell killing. Hydrogel-based composite nanozyme can especially accelerate wound healing and disinfection.

Despite the reporting of various composite nanozymes with enhanced ROS ability and superior therapeutic applications, their translation from the laboratory to market is yet to be achieved. Hence, composite nanozymes need to be engineered to overcome the challenges discussed in Section 6. As this field is still in its infancy and evolving, we expect that the following new paradigms in engineering composite nanozymes could contribute toward addressing these challenges in the near future. 1) The catalytic efficiency of the nanoenzyme could be improved by modifying its lattice (spatial) structure so as to increase active catalytic centers, defect-rich active edges or oxygen vacancies etc. 2) In order to restrict the intrinsic antagonistic catalytic activity of a composite nanozyme, its exact molecular mechanism for electron movements within the nano-composite may be studied toward identifying specific inhibitors that could suppress the antagonistic catalysis. For example, the carbonyl groups on the carbon nanotubes are active sites for their POD mimic activity, while hydroxyl and carboxyl groups act as competitive sites. A study indicated that oxygenated group–enriched carbon nanotubes (o-CNTs) could be modified to show POD activity by blocking the carboxyl group and hydroxyl group. The modified o-CNTs were proved to demonstrate improved antibacterial effects when used in disinfection (Karim et al., 2018). 3) To solve the problem of less specificity and off-target activity, stimuli-responsive nanozymes (such as pH-responsive, hypoxia-responsive, light-responsive, and ultrasonic (US) responsive) could be developed to control the nanozyme’s activation and inactivation. An US switchable nanoplatform (Pd@Pt-T790) system was proposed for the controllable generation of catalytic oxygen and sonosensitizer-mediated reactive oxygen species by ultrasound activation, thereby overcoming the hypoxia-associated barrier and augmenting sonodynamic therapy efficacy. Modification of T790 onto Pd@Pt could block the catalase mimic of Pd@Pt, and upon US irradiation, the nanozyme activity was effectively recovered to catalyze the decomposition of endogenous H_2_O_2_ into O_2_. Such “blocking and activating” enzyme activity was specifically important for decreasing the adverse effects and toxicity of nanozymes on normal cells and has the potential to realize active, controllable, and disease loci–specific composite nanozyme (Sun D. et al., 2020). To overcome the working pH restriction, one strategy has been to use some trigger to accelerate the catalytic action of composite nanozymes at biological pH. From this stand point citrate coated Fe_3_O_4_ NP by utilizing adenosine triphosphate disodium salt (ATP) was used as a synergistic agent to make this nanozyme work at neutral pH as discussed in Section 3.2 (Vallabani et al., 2020). Furthermore for selectivity and specificity, ligand attachment to the nanocomposite could be beneficial for guiding the nanozyme to target cells such as ferritin attachment to nitrogen-doped porous carbon nanospheres, which guides nanocomposite into lysosomes and boosts reactive oxygen species generation in a tumor-specific manner (Fan et al., 2018). In addition, improving the catalytic specificity of nanozymes through artificial molecular imprinting is also a way to realize selectivity. 4) To address the dilemma of biosafety, surface modification or coating is one of the strategies, so it is critical to carefully select an appropriate modifying or coating agent and equip the composite nanozymes for increased biosafety. Also, externally applied H_2_O_2_ toxicity could be avoided by the rational design of cascade nanozymes utilizing *in situ* produced substrates such as H_2_O_2_ and generating ROS. Yan et al. reported an H_2_O_2_-free cascade reaction system by integrating CaO_2_ and hemin-loading graphene (H-G) into alginate (CaO_2_/H-G@alginate). In this nanocomposite, CaO_2_ reacts with the water infiltrated into depots from the interstitial tissues to produce calcium hydroxide [Ca(OH)_2_] and H_2_O_2_. Second, H-G decomposed H_2_O_2_ into hROS. The alginate depots not only offer a confined environment for enhanced reaction efficiency but also improve biocompatibility (Yan et al., 2013). Future research may also use various computational tools and molecular simulation software to help elucidate the catalytic reaction mechanism of nanozymes and establish a structure–function relationship toward designing better nanozymes. Taken together, the pro-oxidative potential of composite nanozymes can be enhanced and harnessed to produce cost-effective, biocompatible, and safe therapeutic agents.
