# Supplementary figures and images for: Recent Trends in Composite Nanozymes and Their Pro-Oxidative Role in Therapeutics
Source: Front Bioeng Biotechnol. 2022 May 30;10:880214. doi: 10.3389/fbioe.2022.880214 (PMC9197165; doi:10.3389/fbioe.2022.880214)

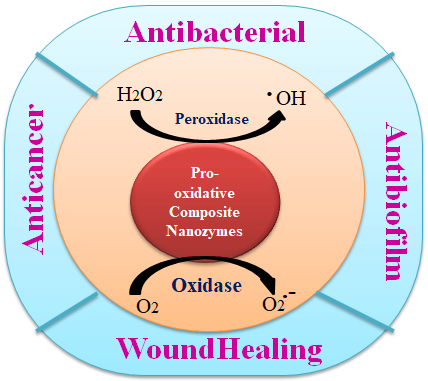

Supplement: Supplementary file 1 [file Image1.TIF]
